# Intraoperative electroencephalogram patterns as predictors of postoperative delirium in older patients: a systematic review and meta-analysis

**DOI:** 10.3389/fnagi.2024.1386669

**Published:** 2024-05-13

**Authors:** Valery V. Likhvantsev, Levan B. Berikashvili, Anastasia V. Smirnova, Petr A. Polyakov, Mikhail Ya Yadgarov, Nadezhda D. Gracheva, Olga E. Romanova, Irina S. Abramova, Maria M. Shemetova, Artem N. Kuzovlev

**Affiliations:** ^1^Federal Research and Clinical Centre of Intensive Care Medicine and Rehabilitology, Department of Clinical Trials, Moscow, Russia; ^2^Department of Anesthesiology, First Moscow State Medical University, Moscow, Russia; ^3^Department of Anesthesiology, City Clinical Oncological Hospital No. 1, Moscow, Russia

**Keywords:** postoperative delirium, intraoperative EEG, burst suppression, systematic review, meta-analysis, surgery, anesthesia

## Abstract

**Background:**

Postoperative delirium (POD) significantly affects patient outcomes after surgery, leading to increased morbidity, extended hospital stays, and potential long-term cognitive decline. This study assessed the predictive value of intraoperative electroencephalography (EEG) patterns for POD in adults.

**Methods:**

This systematic review and meta-analysis followed the PRISMA and Cochrane Handbook guidelines. A thorough literature search was conducted using PubMed, Medline, and CENTRAL databases focusing on intraoperative native EEG signal analysis in adult patients. The primary outcome was the relationship between the burst suppression EEG pattern and POD development.

**Results:**

From the initial 435 articles identified, 19 studies with a total of 7,229 patients were included in the systematic review, with 10 included in the meta-analysis (3,705 patients). In patients exhibiting burst suppression, the POD incidence was 22.1% vs. 13.4% in those without this EEG pattern (p=0.015). Furthermore, an extended burst suppression duration associated with a higher likelihood of POD occurrence (*p* = 0.016). Interestingly, the burst suppression ratio showed no significant association with POD.

**Conclusions:**

This study revealed a 41% increase in the relative risk of developing POD in cases where a burst suppression pattern was present. These results underscore the clinical relevance of intraoperative EEG monitoring in predicting POD in older patients, suggesting its potential role in preventive strategies.

**Systematic Review Registration:**

This study was registered on International Platform for Registered Protocols for Systematic Reviews and Meta-Analyses: INPLASY202420001, https://doi.org/10.37766/inplasy2024.2.0001.

## 1 Introduction

Postoperative delirium (POD) emerges as a multifaceted organic cerebral syndrome, presenting itself as a neuropsychiatric complication following surgical procedures (World Health Organisation, 1994). Marked by abrupt shifts in attention, cognition, and consciousness, POD poses a considerable challenge in perioperative care (O'Regan et al., 2013; Berikashvili et al., 2023). Its implications include heightened patient morbidity, prolonged hospitalization, and an elevated susceptibility to long-term cognitive decline, especially in older patients (Yan et al., 2023).

Given the organic underpinnings of postoperative delirium, monitoring brain activity through electroencephalography (EEG) during surgery emerges as a valuable approach for predicting this complication (Sun et al., 2020). Consequently, recent European guidelines on postoperative delirium management advocate for intraoperative monitoring of anesthesia depth and EEG patterns, specifically the burst suppression pattern, despite the limited quality of evidence supporting this recommendation (Aldecoa et al., 2023).

While a recent meta-analysis suggests that anesthesia guided by bispectral index (BIS) has not significantly reduced the incidence of postoperative delirium (Chew et al., 2022), it is crucial to recognize that excessively deep anesthesia remains a critical risk factor for its development (Evered et al., 2021). The pathological processes induced by overly deep anesthesia, contributing to the clinical manifestation of postoperative delirium, could potentially be discerned through the identification of EEG burst suppression patterns. Moreover, various other EEG signal characteristics may serve as potential predictors of postoperative delirium (Baron Shahaf et al., 2023; Khalifa et al., 2023; Kinoshita et al., 2023). Further research and exploration of EEG monitoring techniques hold promise in refining our understanding and prediction of postoperative delirium in clinical practice.

This systematic review and meta-analysis aim to assess the predictive value of intraoperative EEG for postoperative delirium in adults.

## 2 Materials and methods

This study was conducted in accordance with the Preferred Reporting Items for Systematic Reviews and Meta-Analyses (PRISMA) (Liberati et al., 2009) and Cochrane Handbook guidelines (Higgins et al., 2019). The meta-analysis protocol was registered with the International Platform for Registered Protocols for Systematic Reviews and Meta-Analyses (INPLASY) under registration number INPLASY202420001, https://doi.org/10.37766/inplasy2024.2.0001. The completed PRISMA checklist is presented in Supplementary Table S1.

### 2.1 Search strategy

A comprehensive systematic search was conducted to identify relevant studies published between January 1, 2003, and October 23, 2023. This search encompassed databases such as PubMed, Medline, and the Cochrane Central Register of Controlled Trials (CENTRAL) and was performed by three independent researchers. Additionally, the authors employed forward and backward snowballing methods [Litmaps service (Literature Map Software for Lit Reviews and Research and Litmaps, 2024)]. We did not restrict the search by language. The detailed search strategy and queries are available in Supplementary Appendix A.

### 2.2 Eligibility criteria and study selection

After the automatic exclusion of duplicate records, three researchers independently screened the remaining studies for eligibility based on their titles and abstracts utilizing the PICOS criteria (detailed in Supplementary Appendix B). We focused on prospective and retrospective observational studies that explored predictors of POD using intraoperative native EEG signal analysis in adult patients. The final inclusion in this study was determined after a thorough full-text article analysis. Studies were excluded if they met one of the following criteria: (1) were review articles, case reports or letters to the editors; (2) followed EEG-guided anesthesia; (3) reported no outcome data; (4) utilized non-intraoperative EEG; or (5) evaluated the BIS.

Any disagreements were resolved by consultation with the involvement of the supervisor until a consensus was reached.

### 2.3 Outcome measures and data extraction

For this review, a dedicated data collection form was developed. This form was utilized independently by three authors to independently assess the full manuscripts and supplemental or additional files of all included studies and extract the data. The following data were extracted: study design, sample size, first author, publication year, journal name, POD assessment method, study setting, participant age and sex, American Society of Anesthesiologists (ASA) score, type of anesthesia used, duration of surgery and anesthesia, length of intensive care unit (ICU) and hospital stay, intraoperative EEG timing, and the types and characteristics of EEG patterns in both the POD and non-POD groups. After independent data extraction, the researchers consulted with each other to identify disagreements and reach consensus through discussion.

In instances where the data were presented solely in graphical format, numerical values were extracted using the WebPlotDigitizer tool (Rohatgi, 2010). For studies presenting continuous data in non-standard formats (e.g., median, interquartile range, or 95% confidence intervals), we employed established methods to calculate the mean ± standard deviation (SD). These methods included the statistical techniques proposed by Wan et al. (2014) and Luo et al. (2018), as well as the Cochrane Handbook recommendations 6.5.2.2 and 6.5.2.5 (Higgins and Green, 2011).

This meta-analysis specifically focused on the burst suppression pattern in EEG signals, examining its duration, ratio, and presence (incidence). In this meta-analysis, the BSR is defined as the time EEG shows BSP divided by the total EEG monitoring time. This differs from the clinical BSR, typically a time-varying percentage of BSP over a short period. Our BSR calculation reflects the overall incidence of burst suppression during the entire EEG session, providing a distinct measure for our analysis.

### 2.4 Data analysis and synthesis

In this meta-analysis, STATA 17.0 (StataCorp LLC, Texas, US) was used for both calculations and visualizations. We assessed interstudy heterogeneity using the I-squared (I^2^) statistic and the Cochrane Q test. Standardized mean differences (SMDs) with 95% confidence intervals (CIs) were calculated for quantitative data. In accordance with the Cochrane handbook guidelines, SMDs were categorized into effect sizes: small (< 0.40), moderate (0.40 to 0.70), and large (>0.70) (Higgins and Green, 2011). For categorical outcomes, logarithmic odds ratios (log ORs) and 95% CIs were determined.

A fixed-effects inverse-variance model was applied in cases of low statistical heterogeneity (I^2^ < 60% and *p* > 0.05), while a random-effects model [restricted maximum likelihood (REML)] was used for I^2^ ≥ 60% and/or *p* < 0.05. Statistical significance was set at *p* < 0.05.

The diagnostic accuracy of burst suppression presence was evaluated through pooled metrics, sensitivity, specificity, and positive and negative likelihood ratios, along with the summary receiver operating characteristic (SROC) area under the curve (AUC), employing the 'midas' module in STATA 17.0 (Dwamena, 2007). All the EEG patterns were categorized into six distinct groups based on the specific characteristics of the EEG signals being studied: (1) wave patterns (alpha, beta, delta, theta), (2) burst suppression pattern, and (3) unclassified patterns (Supplementary Table S6). We calculated a weighted average AUCs for the wave patterns.

### 2.5 Internal validity and risk of bias assessment

The internal validity and risk of bias were assessed by two independent reviewers using the “Tool to assess risk of bias in cohort studies” contributed by the CLARITY Group at McMaster University (CLARITY-group. Tool to Assess Risk of Bias in Case Control Studies Hamilton, 2023). An explanation of the risk of bias assessment is presented in Supplementary Table S2. The results were visualized using the “risk-of-bias visualization tool” (McGuinness and Higgins, 2021). Publication bias and small-study effects were assessed using Egger's test and funnel plot analysis.

The certainty of evidence was assessed with the GRADE systematic approach (Guyatt et al., 2008).

### 2.6 Sensitivity analysis

For sensitivity analysis and a more convenient way of comparing effect sizes, direct mean difference (MD), odds ratio (OR) and risk ratio (RR) values were additionally calculated and analyzed.

## 3 Results

### 3.1 Study characteristics

**Figure 1:**
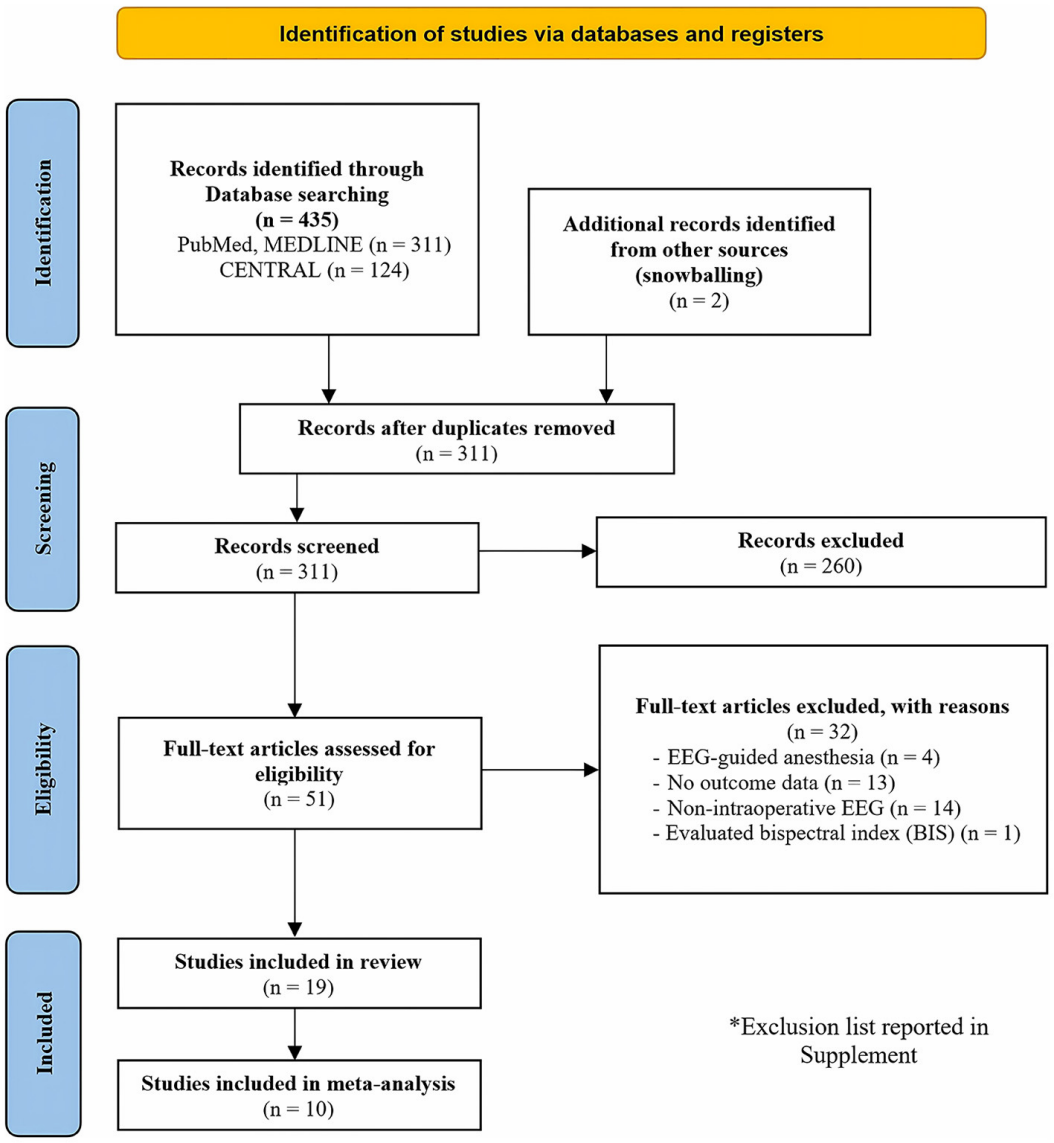
PRISMA flow diagram for study selection.

In the initial search, a total of 435 articles were identified. Following an abstract screening process, 51 articles were selected for full-text evaluation. After careful reading of the full-text articles, 32 studies were excluded (Supplementary Table S3). Ultimately, this systematic review included 19 studies published between 2015 and 2023 (Soehle et al., 2015; Fritz et al., 2016; Hesse et al., 2019; Momeni et al., 2019; Pedemonte et al., 2020; Jung et al., 2021; Koch et al., 2021, 2023; Lele et al., 2022; Li et al., 2022; Lutz et al., 2022; Röhr et al., 2022; Windmann et al., 2022; Baron Shahaf et al., 2023; Dragovic et al., 2023; Khalifa et al., 2023; Kinoshita et al., 2023; Reese et al., 2023; Ostertag et al., 2024). Additionally, 10 of these articles were included in the meta-analysis (Soehle et al., 2015; Hesse et al., 2019; Pedemonte et al., 2020; Jung et al., 2021; Koch et al., 2021, 2023; Lele et al., 2022; Lutz et al., 2022; Röhr et al., 2022; Ostertag et al., 2024). A flowchart illustrating the study selection process is presented in Figure 1.

In this systematic review, a total of 7,229 patients were analyzed, comprising 1,370 patients with POD (POD+) and 5,859 without (POD-). The data extracted from the included articles are detailed in Supplementary Tables S4–S6. The characteristics of the studies included in the meta-analysis are summarized in Table 1. In the meta-analysis of 3,705 patients, 18.8% (696) developed POD. The meta-analysis included five prospective observational studies, two *post-hoc* analyses of randomized trials, two retrospective observational studies, and one *post-hoc* analysis of a prospective observational trial. The mean age ranged from 59.8 to 72.9 years, and the proportion of patients with ASA III-V varied from 23.7% to 95.6% (Table 1).

**Table 1:** Characteristics and description of the 10 trials included in the meta-analysis.

**Study**	**Design**	**Journal**	**Sample size (All)**	**POD+ (N)**	**POD−(N)**	**Sex, male %**	**Age, mean**	**ASA III-V, %**	**POD assessment method**	**EEG pattern**
Lele et al. (2022)	ROS	J Neurosurg Anesthesiol	112	10	102	54.5	59.8	ND	CAM	PBS; DBS
Jung et al. (2021)	POS	Medicine (Baltimore)	80	13	67	59.0	66.3	45.0	3D-CAM	DBS
Pedemonte et al. (2020)	ROS	Anesthesiology	159	23	136	69.0	70.1	95.6	CAM	PBS
Ostertag et al. (2024)	POS p-h	Anesthesiology	169	32	137	75.1	61.7	23.7	CAM-ICU	PBS
Soehle et al. (2015)	POS	BMC Anesthesiol	81	26	55	70.4	72.9	ND	CAM-ICU	DBS
Lutz et al. (2022)	POS	J Clin Anesth	116	25	91	75.9	62.6	26.7	CAM-ICU, RASS	PBS
Hesse et al. (2019)	POS	Br J Anaesth	626	125	501	61.0	69.7	48.2	CAM-ICU	PBS
Koch et al. (2021)	POS	Anesth Analg	237	41	196	53.0	72.8	37.0	DSM V	DBS
Koch et al. (2023)	RCT p-h	Front Aging Neurosci	1058	198	860	54.0	69.7	47.7	DSM IV	BSR; DBS
Röhr et al. (2022)	RCT p-h	Front Aging Neurosci	1067	203	864	ND	69.7	ND	DSM IV	BSR

ROS, retrospective observational study; POS, prospective observational study; POS p-h, *post hoc* prospective observational study; RCT p-h, randomized controlled trial *post hoc*; PBS, presence of burst suppression; BSR, burst suppression ratio; DBS, duration of burst suppression (min); POD, postoperative delirium; CAM, Confusion Assessment Method; ASA, American Society of Anesthesiologists; ND, no data.

### 3.2 Presence of burst suppression

**Figure 2:**
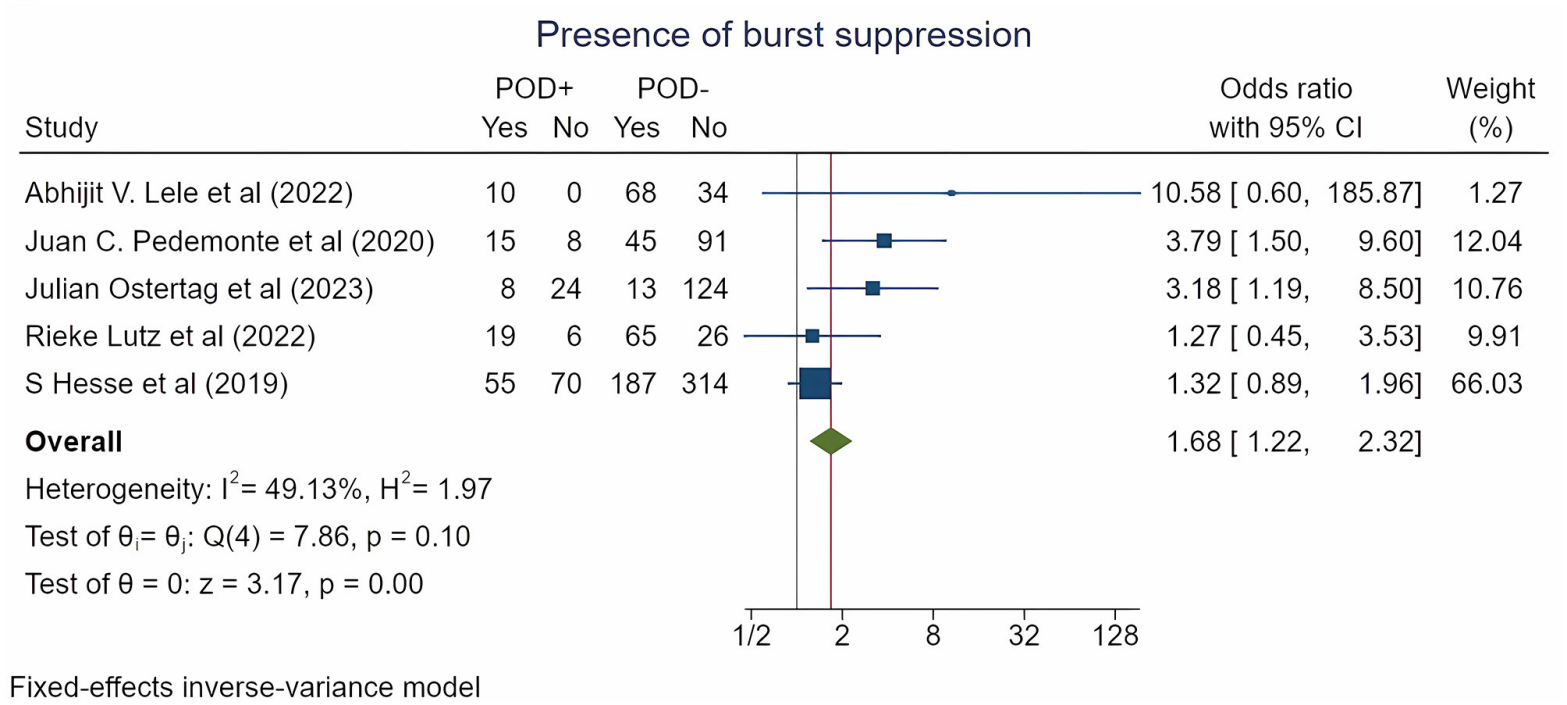
Forest plot for burst suppression incidence by POD type. The plot displays the studies, sample sizes, odds ratios, confidence intervals (CIs), and *p-*values. The size of the squares indicates the weight of the studies (considering sample size and standard deviations); the diamond represents the pooled odds ratio with CIs.

According to a meta-analysis of five studies encompassing 1,182 patients and reporting the burst suppression episodes on EEG, the incidence was significantly higher in patients who experienced POD (22.1% vs. 13.4%) [OR = 1.68 (1.22; 2.32), *p* = 0.015; log OR = 0.52 (0.2; 0.84), *p* = 0.002; RR = 1.41 (1.1; 1.8), *p* = 0.006] (Figure 2, Table 2, Supplementary Figures S1-S3).

**Table 2:** Outcomes and sensitivity analysis.

**Outcome**	**Trials**	**POD+, N**	**POD-, N**	**Overall effects (95% CI)**	***p*-value for overall effects**	***p*-value for heterogeneity**	**I^2^, %**	***p*-value for publication bias**
Burst suppression presence, *n*	5	215	967	Log OR: 0.52 (0.20; 0.84)	0.002	0.097	49.13	For log OR: 0.031
				OR: 1.68 (1.22; 2.32)	0.015	0.097	49.13	
				RR: 1.41 (1.10; 1.80)	0.006	0.021	70.04	
Duration of burst suppression, min	5	288	1280	SMD: 0.36 (0.23; 0.49)	< 0.001	0.085	51.07	For SMD: 0.327
				MD: 15.86 (3.02; 28.70)	0.016	0.031	60.42	
Burst suppression ratio	2	401	1724	SMD: 0.07 (−0.04; 0.18)	0.215	0.600	0	For SMD: 0.600
				MD: 0.01 (−0.0; 0.02)	0.217	0.592	0	

POD, postoperative delirium; MD, mean difference; SMD, standardized mean difference; OR, odds ratio; Log OR, logarithm of the odds ratio; RR, risk ratio; CI, confidence interval; NA, not applicable.

**Figure 3:**
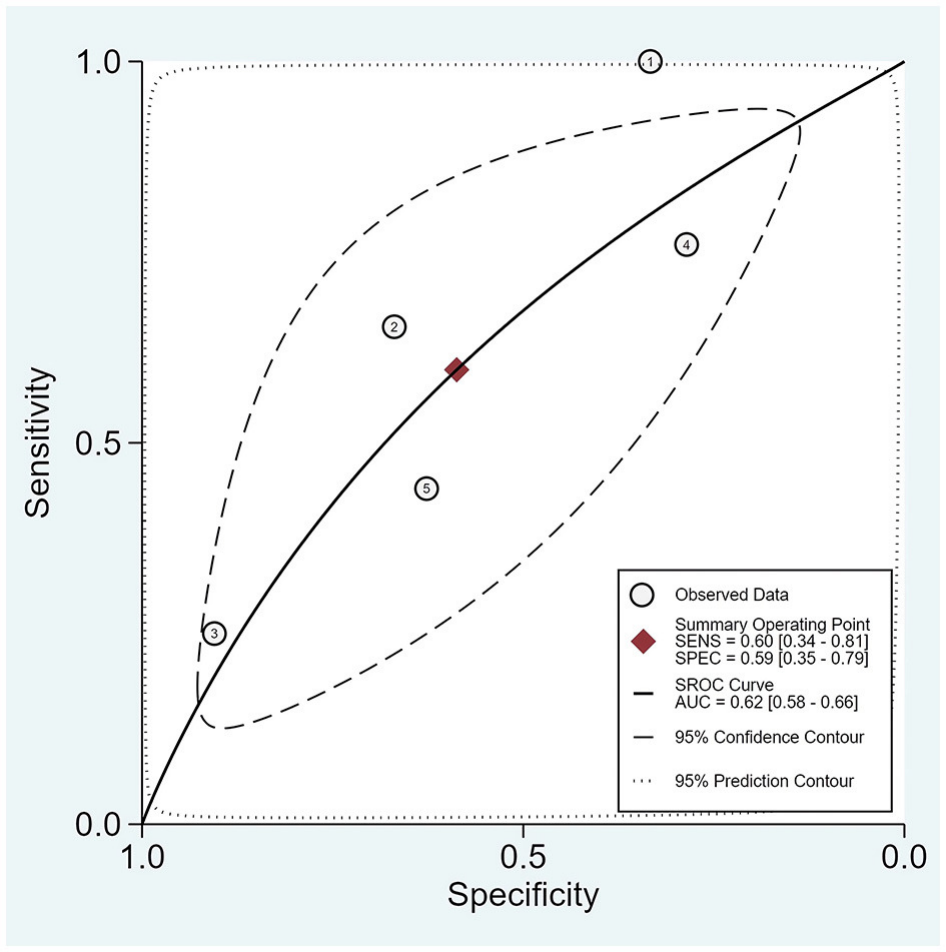
SROC for the presence of burst suppression. The plot displays the summary ROC curve (SROC) and presents AUC with 95% confidence interval (CI), 95% prediction and confidence contours, sensitivity, and specificity with CIs.

The area under the SROC curve for the presence of burst suppression was 0.620 (0.580; 0.660) (Figure 3), with a pooled sensitivity of 0.60 (0.34; 0.81) and a specificity of 0.59 (0.35; 0.79) (Supplementary Figure S4).

### 3.3 Duration of burst suppression

According to a pooled analysis of five studies reporting this outcome and involving 1,568 patients, patients who experienced POD had a significantly extended duration of burst suppression on EEG [MD = 15.86 (3.02; 28.70) minutes, *p* = 0.016; SMD = 0.36 (0.23; 0.49), *p* < 0.001] (Table 2, Supplementary Figures S5, S6).

### 3.4 Burst suppression ratio

In a pooled analysis of two studies involving 2,125 patients and reporting the burst suppression ratio on EEG, we found no significant relationship with POD [MD = 0.007 (−0.004; 0.018), *p* = 0.217; SMD = 0.07 (−0.04; 0.18), *p* = 0.215] (Table 2, Supplementary Figures S7, S8).

### 3.5 Weighted average AUC values

The weighted average AUC values for the alpha (0.676), beta (0.670), delta (0.660), and theta (0.685) wave patterns were determined from studies included in the systematic review (Supplementary Table S6).

### 3.6 Risk of bias and GRADE assessment

The overall risk of bias of the 10 enrolled studies was judged as ‘low' in three studies and ‘some concerns' in 7 studies (Supplementary Figure S9). The main sources of bias identified were the lack of matching for confounding variables and inconsistency in exposure assessment. Egger's test and funnel plot analysis did not reveal small-study effects for the majority of the study outcomes (Supplementary Figure S10). Publication bias was statistically significant for the presence of burst suppression (Table 2, Supplementary Figure S10). According to the GRADE approach, a moderate level of evidence supported the association between duration of burst suppression and POD, while evidence for the incidence of burst suppression was considered very low (Supplementary Table S7).

## 4 Discussion

### 4.1 Key findings

This is the first systematic review and meta-analysis to evaluate various aspects of burst suppression pattern of EEG signals as prognostic factors of postoperative delirium in older adults. The meta-analysis revealed that the duration of burst suppression is extended in patients who have developed postoperative delirium. In addition, the occurrence of a burst suppression pattern was associated with a 1.4-fold increased risk of developing POD (a relative risk increases of 41%). However, the SROC value for presence of burst suppression pattern was only 0.62, indicating that this pattern was a satisfactory prognostic factor for POD development. Investigations into other various wave patterns also demonstrated similar satisfactory predictive capabilities (the average weighted area under the curve (AUC) varied from 0.660 to 0.685).

### 4.2 Relationship with previous studies

This meta-analysis is the first to comprehensively examine the relationship between various EEG patterns and POD. Despite finding an association between the presence of the burst-suppression EEG pattern and the development of postoperative delirium, these results were not consistent across all studies (Hesse et al., 2019; Lele et al., 2022; Lutz et al., 2022). However, since the primary cause of this pattern is excessively deep anesthesia, our findings likely explain the results of the meta-analysis by Sumner M et al., which showed that EEG-guided anesthesia is associated with a reduced risk of POD (Sumner et al., 2023). Additionally, our results regarding the duration of burst suppression and its link to POD may also elucidate the findings of Sumner et al. (2023). In the systematic review conducted by Bruzzone et al. (2023) a detailed analysis was presented showing associations between specific intraoperative EEG parameters and the development of POD. The study found that increased magnitude and longer durations of EEG suppression, alongside a reduction in higher frequency activity, were significant indicators of POD risk. Furthermore, it was noted that an increased incidence and duration of BSR and lower BIS values are also predictive of POD development. Nonetheless, several studies have not demonstrated an association between this parameter and POD (Koch et al., 2021, 2023; Lele et al., 2022).

### 4.3 Significance of the study findings

The significance of our study findings can be understood in two key aspects.

First, this inaugural meta-analysis investigated the effects of deep anesthesia, characterized by the presence of a burst suppression pattern on EEG signals, on the incidence of POD. Identifying statistically significant differences in burst suppression pattern, in terms of incidence and duration, between patients with and without POD is promising for advancing our understanding of postoperative delirium prediction and prevention strategies.

Second, our meta-analysis revealed a lack of comprehensive data on intraoperative EEG patterns. A major challenge in studying the prognostic qualities of EEG patterns is the absence of a standardized set of metrics for evaluation. This limitation hinders the possibility of conducting extensive meta-analyses. Additionally, the lack of standardization in the aspects of recording periods further diminishes the quality of the existing evidence. Despite current recommendations advocating for perioperative EEG monitoring, especially concerning the burst suppression pattern, the shift in clinical practices necessitates robust, high-quality evidence to adhere to evidence-based clinical decision-making principles. Our study provides, for the first time, high-quality evidence supporting the integration of perioperative EEG monitoring in the diagnostic framework for POD. Our results align with and reinforce the latest guidelines, thereby enhancing the evidence quality in this domain. In summary, this research not only highlights the clinical value of intraoperative EEG monitoring but also underscores the need for further high-quality studies to strengthen the evidence base in this area.

### 4.4 Strengths and limitations

To the best of our knowledge, this is the first meta-analysis in this field. Despite the inability to conduct a meta-analysis on a wide range of other diverse EEG patterns, we successfully grouped them based on their relation to one of the EEG waves (alpha, beta, theta, delta) and calculated the weighted average AUCs for each group. While the values of the weighted average AUCs were comparable, a more in-depth exploration of the existing patterns is of significant scientific interest. All studies included in the meta-analysis exhibited a moderate or low risk of bias, enhancing the quality of the obtained results.

Nevertheless, it is important to acknowledge the limitations in this analysis. The high heterogeneity among the studies presented in this work complicates the process of data synthesis resulting from the analysis. Additionally, the burst suppression ratio did not demonstrate statistical significance in predicting POD, which may be attributed to the limited availability of data and studies examining this pattern. We cannot overlook that the development of POD is influenced by a multitude of factors, such as patients' medical histories, characteristics of the intraoperative period, and surgical complications during the perioperative period. To gain a more precise understanding of the nature of POD onset, these factors must be considered in the analysis. We also observed significant publication bias for burst suppression incidence, which suggests that the findings should be interpreted with caution. Moreover, using EEG to predict POD during the intraoperative period has several limitations. Individual variability in brain activity, influenced by factors like age and neurological history, can complicate EEG signal interpretation. The intraoperative setting introduces artifacts from surgical and medical equipment, challenging the clarity and reliability of EEG data. Additionally, anesthetic agents alter EEG patterns, necessitating careful consideration of their effects in POD prediction. Standardization issues in EEG protocol, such as electrode placement and signal processing, further limit the consistency and generalizability of findings across studies.

### 4.5 Future studies and prospects

A comprehensive analysis of the conducted studies has highlighted the need for further exploration of the burst suppression pattern through the execution of high-quality prospective observational studies dedicated to its examination. The substantial heterogeneity among the studied patterns raises considerations about standardizing the methods for assessing individual types of EEG waves, potentially facilitating subsequent meta-analyses to assess the prognostic significance of these parameters in predicting postoperative delirium.

## 5 Conclusion

This systematic review and meta-analysis demonstrated that the occurrence of the burst suppression pattern on EEG was associated with a 41% increase in the relative risk of POD development in older patients. Additionally, the duration of burst suppression was also extended in patients with POD. Our research provides strong evidence for expanding the use of intraoperative EEG monitoring in current guidelines. This study highlights EEG's value in improving perioperative care by assessing brain activity and detecting delirium risk. Our findings advocate for integrating EEG monitoring into routine intraoperative procedures to enhance patient outcomes and support more personalized anesthetic management.

## Data availability statement

The original contributions presented in the study are included in the article/Supplementary material, further inquiries can be directed to the corresponding author.

## Author contributions

VL: Conceptualization, Methodology, Project administration, Writing – original draft, Writing – review & editing. LB: Conceptualization, Formal analysis, Methodology, Writing – original draft, Writing – review & editing. AS: Data curation, Investigation, Writing – original draft, Writing – review & editing. PP: Formal analysis, Methodology, Writing – original draft, Writing – review & editing. MY: Formal analysis, Writing – original draft, Writing – review & editing. NG: Data curation, Investigation, Writing – original draft, Writing – review & editing. OR: Data curation, Investigation, Writing – original draft, Writing – review & editing. IA: Writing – original draft, Writing – review & editing. MS: Project administration, Writing – original draft, Writing – review & editing. AK: Project administration, Writing – original draft, Writing – review & editing.
