# Supplementary material for: Intraoperative electroencephalogram patterns as predictors of postoperative delirium in older patients: a systematic review and meta-analysis
Source: Front Aging Neurosci. 2024 May 13;16:1386669. doi: 10.3389/fnagi.2024.1386669 (PMC11128674; doi:10.3389/fnagi.2024.1386669)
Supplement: Supplementary file 1 [file Data_Sheet_1.docx]

Supplementary Material

# Supplementary Appendix A

Search strategy for each database.

**PubMed, MEDLINE:** (EEG [tiab] OR electroencephalography [tiab] OR electroencephalogical [tiab]) AND delirium [tiab] NOT ("electroencephalography-guided" OR children OR pediatric OR paediatric OR “COVID-19” [tiab] OR coronavirus [tiab] OR “EEG Guidance”) AND ("2003/01/01"[PDAT]: "2023/10/23"[PDAT])

**Cochrane CENTRAL:** (Electroencephalography OR EEG) AND delirium NOT (electroencephalography-guided OR children OR pediatric OR paediatric OR COVID-19 OR coronavirus OR EEG Guidance)

# Supplementary Appendix B

PICOS criteria.

**Population:** adult patients following surgery. **Intervention (exposure):** presence of intraoperative EEG pattern associated with postoperative delirium. **Comparator:** absence of intraoperative EEG pattern associated with postoperative delirium. **Outcomes:** postoperative delirium. **Study design:** prospective and retrospective observational studies, post hoc analyses of randomized controlled trials. **Time range:** Jan 01, 2003-October 23, 2023.

# Table S1. PRISMA Checklist.

| **Section and Topic** | **Item #** | **Checklist item** | **Location where item is reported** |
| --- | --- | --- | --- |
| **TITLE** | | |  |
| Title | 1 | Identify the report as a systematic review. | Title page |
| **ABSTRACT** | | |  |
| Abstract | 2 | See the PRISMA 2020 for Abstracts checklist. | Abstract |
| **INTRODUCTION** | | |  |
| Rationale | 3 | Describe the rationale for the review in the context of existing knowledge. | Introduction |
| Objectives | 4 | Provide an explicit statement of the objective(s) or question(s) the review addresses. | Introduction |
| **METHODS** | | |  |
| Eligibility criteria | 5 | Specify the inclusion and exclusion criteria for the review and how studies were grouped for the syntheses. | Methods |
| Information sources | 6 | Specify all databases, registers, websites, organisations, reference lists and other sources searched or consulted to identify studies. Specify the date when each source was last searched or consulted. | Methods |
| Search strategy | 7 | Present the full search strategies for all databases, registers and websites, including any filters and limits used. | Methods, Supplement |
| Selection process | 8 | Specify the methods used to decide whether a study met the inclusion criteria of the review, including how many reviewers screened each record and each report retrieved, whether they worked independently, and if applicable, details of automation tools used in the process. | Methods |
| Data collection process | 9 | Specify the methods used to collect data from reports, including how many reviewers collected data from each report, whether they worked independently, any processes for obtaining or confirming data from study investigators, and if applicable, details of automation tools used in the process. | Methods |
| Data items | 10a | List and define all outcomes for which data were sought. Specify whether all results that were compatible with each outcome domain in each study were sought (e.g. for all measures, time points, analyses), and if not, the methods used to decide which results to collect. | Methods |
|  | 10b | List and define all other variables for which data were sought (e.g. participant and intervention characteristics, funding sources). Describe any assumptions made about any missing or unclear information. | Methods |
| Study risk of bias assessment | 11 | Specify the methods used to assess risk of bias in the included studies, including details of the tool(s) used, how many reviewers assessed each study and whether they worked independently, and if applicable, details of automation tools used in the process. | Methods |
| Effect measures | 12 | Specify for each outcome the effect measure(s) (e.g. risk ratio, mean difference) used in the synthesis or presentation of results. | Methods |
| Synthesis methods | 13a | Describe the processes used to decide which studies were eligible for each synthesis (e.g. tabulating the study intervention characteristics and comparing against the planned groups for each synthesis (item #5)). | Methods |
|  | 13b | Describe any methods required to prepare the data for presentation or synthesis, such as handling of missing summary statistics, or data conversions. | Methods |
|  | 13c | Describe any methods used to tabulate or visually display results of individual studies and syntheses. | Methods |
|  | 13d | Describe any methods used to synthesize results and provide a rationale for the choice(s). If meta-analysis was performed, describe the model(s), method(s) to identify the presence and extent of statistical heterogeneity, and software package(s) used. | Methods |
|  | 13e | Describe any methods used to explore possible causes of heterogeneity among study results (e.g. subgroup analysis, meta-regression). | Methods |
|  | 13f | Describe any sensitivity analyses conducted to assess robustness of the synthesized results. | Methods |
| Reporting bias assessment | 14 | Describe any methods used to assess risk of bias due to missing results in a synthesis (arising from reporting biases). | Methods |
| Certainty assessment | 15 | Describe any methods used to assess certainty (or confidence) in the body of evidence for an outcome. | Methods |
| **RESULTS** | | |  |
| Study selection | 16a | Describe the results of the search and selection process, from the number of records identified in the search to the number of studies included in the review, ideally using a flow diagram. | Results, Fig. 1 |
|  | 16b | Cite studies that might appear to meet the inclusion criteria, but which were excluded, and explain why they were excluded. | Results |
| Study characteristics | 17 | Cite each included study and present its characteristics. | Table 1 |
| Risk of bias in studies | 18 | Present assessments of risk of bias for each included study. | Supplement |
| Results of individual studies | 19 | For all outcomes, present, for each study: (a) summary statistics for each group (where appropriate) and (b) an effect estimate and its precision (e.g. confidence/credible interval), ideally using structured tables or plots. | Results, Table 2 |
| Results of syntheses | 20a | For each synthesis, briefly summarise the characteristics and risk of bias among contributing studies. | Results, Table 2 |
|  | 20b | Present results of all statistical syntheses conducted. If meta-analysis was done, present for each the summary estimate and its precision (e.g. confidence/credible interval) and measures of statistical heterogeneity. If comparing groups, describe the direction of the effect. | Results, Table 2 |
|  | 20c | Present results of all investigations of possible causes of heterogeneity among study results. | Results, Table 2 |
|  | 20d | Present results of all sensitivity analyses conducted to assess the robustness of the synthesized results. | Results, Table 2 |
| Reporting biases | 21 | Present assessments of risk of bias due to missing results (arising from reporting biases) for each synthesis assessed. | Supplement |
| Certainty of evidence | 22 | Present assessments of certainty (or confidence) in the body of evidence for each outcome assessed. | Supplement |
| **DISCUSSION** | | |  |
| Discussion | 23a | Provide a general interpretation of the results in the context of other evidence. | Discussion |
|  | 23b | Discuss any limitations of the evidence included in the review. | Discussion |
|  | 23c | Discuss any limitations of the review processes used. | Discussion |
|  | 23d | Discuss implications of the results for practice, policy, and future research. | Discussion |
| **OTHER INFORMATION** | | |  |
| Registration and protocol | 24a | Provide registration information for the review, including register name and registration number, or state that the review was not registered. | Provided |
|  | 24b | Indicate where the review protocol can be accessed, or state that a protocol was not prepared. | Provided |
|  | 24c | Describe and explain any amendments to information provided at registration or in the protocol. | Provided |
| Support | 25 | Describe sources of financial or non-financial support for the review, and the role of the funders or sponsors in the review. | Provided |
| Competing interests | 26 | Declare any competing interests of review authors. | Provided |
| Availability of data, code and other materials | 27 | Report which of the following are publicly available and where they can be found: template data collection forms; data extracted from included studies; data used for all analyses; analytic code; any other materials used in the review. | Provided |

# Table S2. Risk of bias explanation.

| The following questions are derived from the “Tool to assess risk of bias in cohort studies” contributed by the CLARITY Group at McMaster University:  <https://www.evidencepartners.com/wp-content/uploads/2017/09/Tool-to-Assess-Risk-of-Bias-in-Cohort-Studies.pdf>  The questionnaire divides the cohorts as exposed (in the context of the study - patients with EEG pattern ‘+’) and non-exposed (EEG pattern ‘-’). The examples beneath the questions are intended to clarify the rationale behind answers in each question.  In each question, 4 answers were possible:  1. Definitely yes (low risk of bias)  2. Probably yes (come concerns)  3. Probably no (high risk of bias)  4. Definitely no (very-high risk of bias)  We employed an average bias method to calculate the overall risk of bias. For each study, a mean score was computed, considering the following criteria: DY (Definitely Yes) was assigned a score of 0, PY (Probably Yes) a score of 1, PN (Probably No) a score of 2, and DN (Definitely No) a score of 3. We employed a rounding method for the average score, rounding up except for values of 0.125, 1.125, and 2.125. In addition, we implemented a rule that the overall risk of bias should not differ by more than 1 level from the maximum bias observed across all domains.  ***Q1. Was selection of exposed and non-exposed cohorts drawn from the same population?***  Exposed and unexposed patients drawn for same cohort of patients presenting at same points of care over the same time frame.  ***Q2. Can we be confident in the assessment of exposure?***  The following criteria were evaluated:   - time point of exposure assessment; - how EEG patterns were assessed; - what EEG machine and software were used; - presence of at least two EEG experts.   **Definitely yes:** All criteria of exposure assessment were stated.  **Probably yes:** One criterion of exposure assessment was not stated.  **Probably no:** Some criteria of exposure assessment were not stated.  **Definitely no:** Any criteria of exposure were not stated.  ***Q3. Can we be confident that the outcome of interest was not present at start of study?***  A **definitely yes** was prespecified as the appropriate answer for prospective studies;  A **probably yes** was prespecified as the appropriate answer for retrospective studies.  ***Q4. Did the study match exposed and unexposed for all variables that are associated with the outcome of interest or did the statistical analysis adjust for these prognostic variables?***  **Definitely yes:** All of the important confounding variables were comparable between exposed and unexposed groups.  **Probably yes:** One of some important confounding variables was not similar between exposed and unexposed groups.  **Probably no:** Some of important confounding variables were not similar between exposed and unexposed groups.  **Definitely no:** No data on confounding variables.  ***Q5. Can we be confident in the assessment of the presence or absence of prognostic factors?***  The following criteria were evaluated:  **Age, setting, dementia, premedication, ASA.**  **Definitely yes:** All variables were assessed.  **Probably yes:** One of the prognostic variables was not assessed.  **Probably no:** Some of the prognostic variables was not assessed.  **Definitely no:** No data on prognostic factors presented.  ***Q6. Can we be confident in the assessment of outcome?***  Due to the nature of our outcome (POD), **a definitely yes** was prespecified as the appropriate answer if the validated method of POD assessment was used.  ***Q7. Was the follow up of cohorts adequate?***  Due to the nature of our outcome (POD), **a definitely yes** was prespecified as the appropriate answer.  ***Q8. Were co-Interventions similar between groups?***  The comparability of anesthesia protocols in exposed and unexposed groups was assessed.  **Definitely yes:** Patients in exposed and unexposed groups had similar co-interventions (or there were no co-interventions).  **Probably yes:** There was some difference in co-interventions that probably didn’t affect studied outcome.  **Probably no:** There was some difference in co-interventions that probably affected studied outcome.  **Definitely no:** There was some difference in co-interventions that definitely affected studied outcome (or no data on co-interventions). |
| --- |

# Table S3. Excluded studies with a cause of exclusion.

| **Cause of exclusion** | **Study** |
| --- | --- |
| **EEG-guided anesthesia** | 1. Wang E, Wang L, Ye C, et al. Effect of Electroencephalography Spectral Edge Frequency (SEF) and Patient State Index (PSI)-Guided Propofol-Remifentanil Anesthesia on Delirium After Laparoscopic Surgery: The eMODIPOD Randomized Controlled Trial. J Neurosurg Anesthesiol. 2022;34(2):183-192. doi:10.1097/ANA.0000000000000823 2. Fritz BA, King CR, Ben Abdallah A, et al. Preoperative Cognitive Abnormality, Intraoperative Electroencephalogram Suppression, and Postoperative Delirium: A Mediation Analysis. Anesthesiology. 2020;132(6):1458-1468. doi:10.1097/ALN.0000000000003181 3. Chen YC, Hung IY, Hung KC, et al. Incidence change of postoperative delirium after implementation of processed electroencephalography monitoring during surgery: a retrospective evaluation study. BMC Anesthesiol. 2023;23(1):330. doi:10.1186/s12871-023-02293-9 4. Tang CJ, Jin Z, Sands LP, et al. ADAPT-2: A Randomized Clinical Trial to Reduce Intraoperative EEG Suppression in Older Surgical Patients Undergoing Major Noncardiac Surgery. Anesth Analg. 2020;131(4):1228-1236. doi:10.1213/ANE.0000000000004713 |
| **No outcome data** | 1. Barreto Chang OL, Kreuzer M, Morgen DF, Possin KL, García PS. Ketamine-Associated Intraoperative Electroencephalographic Signatures of Elderly Patients with and Without Preoperative Cognitive Impairment. Anesth Analg. 2022;135(4):683-692. doi:10.1213/ANE.0000000000005875 2. Acker L, Ha C, Zhou J, et al. Electroencephalogram-Based Complexity Measures as Predictors of Post-operative Neurocognitive Dysfunction. Front Syst Neurosci. 2021;15:718769. doi:10.3389/fnsys.2021.718769 3. Fleischmann A, Pilge S, Kiel T, Kratzer S, Schneider G, Kreuzer M. Substance-specific differences in human electroencephalographic burst suppression patterns. Front Hum Neurosci. 2018;12:368. doi:10.3389/fnhum.2018.00368 4. Muhlhofer WG, Zak R, Kamal T, et al. Burst-suppression ratio underestimates absolute duration of electroencephalogram suppression compared with visual analysis of intraoperative electroencephalogram. Br J Anaesth. 2017;118(5):755-761. doi:10.1093/bja/aex054 5. Lapointe AP, Li D, Hudetz AG, Vlisides PE. Microstate analyses as an indicator of anesthesia-induced unconsciousness. Clin Neurophysiol. 2023;147:81-87. doi:10.1016/j.clinph.2023.01.007 6. Fang P pan, Shang Z xiang, Xu J, et al. Contribution of intraoperative electroencephalogram suppression to frailty-associated postoperative delirium: mediation analysis of a prospective surgical cohort. Br J Anaesth. 2023;130(2):e263-e271. doi:10.1016/j.bja.2022.11.002 7. Shao YR, Kahali P, Houle TT, et al. Low Frontal Alpha Power Is Associated With the Propensity for Burst Suppression: An Electroencephalogram Phenotype for a “Vulnerable Brain.” Anesth Analg. 2020;131(5):1529-1539. doi:10.1213/ANE.0000000000004781 8. Plummer GS, Ibala R, Hahm E, et al. Electroencephalogram dynamics during general anesthesia predict the later incidence and duration of burst-suppression during cardiopulmonary bypass. Clin Neurophysiol. 2019;130(1):55-60. doi:10.1016/j.clinph.2018.11.003 9. Vlisides PE, Li D, Maywood M, et al. Electroencephalographic Biomarkers, Cerebral Oximetry, and Postoperative Cognitive Function in Adult Noncardiac Surgical Patients: A Prospective Cohort Study. Anesthesiology. 2023;139(5):568-579. doi:10.1097/ALN.0000000000004664 10. Koch S, Feinkohl I, Chakravarty S, et al. Cognitive Impairment Is Associated with Absolute Intraoperative Frontal α-Band Power but Not with Baseline α-Band Power: A Pilot Study. Dement Geriatr Cogn Disord. 2019;48(1-2):83-92. doi:10.1159/000502950 11. Belletti A, Lee DK, Yanase F, et al. Changes in SedLine-derived processed electroencephalographic parameters during hypothermia in patients undergoing cardiac surgery with cardiopulmonary bypass. Front Cardiovasc Med. 2023;10:1084426. doi:10.3389/fcvm.2023.1084426 12. Ma O, Crepeau AZ, Dutta A, Bliss DW. Anticipating Postoperative Delirium during Burst Suppression Using Electroencephalography. IEEE Trans Biomed Eng. 2020;67(9):2659-2668. doi:10.1109/TBME.2020.2967693 13. Fritz BA, Maybrier HR, Avidan MS. Intraoperative electroencephalogram suppression at lower volatile anaesthetic concentrations predicts postoperative delirium occurring in the intensive care unit. Br J Anaesth. 2018;121(1):241-248. doi:10.1016/j.bja.2017.10.024 |
| **Non-intraoperative EEG** | 1. Mashour GA, Palanca BJA, Basner M, et al. Recovery of consciousness and cognition after general anesthesia in humans. Elife. 2021;10. doi:10.7554/eLife.59525 2. Van Der Kooi AW, Zaal IJ, Klijn FA, et al. Delirium detection using EEG: What and how to measure. Chest. 2015;147(1):94-101. doi:10.1378/chest.13-3050 3. Arai N, Miyazaki T, Nakajima S, et al. The Association between Electroencephalography with Auditory Steady-State Response and Postoperative Delirium. J Pers Med. 2023;13(1). doi:10.3390/jpm13010035 4. Kimchi EY, Neelagiri A, Whitt W, et al. Clinical EEG slowing correlates with delirium severity and predicts poor clinical outcomes. Neurology. 2019;93(13):E1260-E1271. doi:10.1212/WNL.0000000000008164 5. Kim H, McKinney A, Brooks J, Mashour GA, Lee UC, Vlisides PE. Delirium, Caffeine, and Perioperative Cortical Dynamics. Front Hum Neurosci. 2021;15:744054. doi:10.3389/fnhum.2021.744054 6. Numan T, Slooter AJC, van der Kooi AW, et al. Functional connectivity and network analysis during hypoactive delirium and recovery from anesthesia. Clin Neurophysiol. 2017;128(6):914-924. doi:10.1016/j.clinph.2017.02.022 7. Tanabe S, Parker M, Lennertz R, Pearce RA, Banks MI, Sanders RD. Reduced Electroencephalogram Complexity in Postoperative Delirium. Journals Gerontol - Ser A Biol Sci Med Sci. 2022;77(3):502-506. doi:10.1093/gerona/glab352 8. Hut SCA, Dijkstra-Kersten SMA, Numan T, et al. EEG and clinical assessment in delirium and acute encephalopathy. Psychiatry Clin Neurosci. 2021;75(8):265-266. doi:10.1111/pcn.13225 9. Kim SB, Bong SH, Lee JH, Choi TY, Yoon SY, Kim JW. The Usefulness of Quantitative Electroencephalography in Diagnosis and Severity Evaluation of Delirium: A Retrospective Study. Psychiatry Investig. 2023;20(2):144-151. doi:10.30773/pi.2022.0294 10. Yamanashi T, Crutchley KJ, Wahba NE, et al. Evaluation of point-of-care thumb-size bispectral electroencephalography device to quantify delirium severity and predict mortality. Br J Psychiatry. 2022;220(6):322-329. doi:10.1192/bjp.2021.101 11. Guay CS, Kafashan M, Huels ER, et al. Postoperative Delirium Severity and Recovery Correlate with Electroencephalogram Spectral Features. Anesth Analg. 2023;136(1):140-151. doi:10.1213/ANE.0000000000006075 12. Numan T, van den Boogaard M, Kamper AM, et al. Delirium detection using relative delta power based on 1-minute single-channel EEG: a multicentre study. Br J Anaesth. 2019;122(1):60-68. doi:10.1016/j.bja.2018.08.021 13. Yamanashi T, Kajitani M, Iwata M, et al. Topological data analysis (TDA) enhances bispectral EEG (BSEEG) algorithm for detection of delirium. Sci Rep. 2021;11(1):304. doi:10.1038/s41598-020-79391-y 14. Tanabe S, Mohanty R, Lindroth H, et al. Cohort study into the neural correlates of postoperative delirium: the role of connectivity and slow-wave activity. Br J Anaesth. 2020;125(1):55-66. doi:10.1016/j.bja.2020.02.027 |
| **Evaluated bispectral index (BIS)** | 1. Cooter Wright M, Bunning T, Eleswarpu SS, et al. A Processed Electroencephalogram-Based Brain Anesthetic Resistance Index Is Associated with Postoperative Delirium in Older Adults: A Dual Center Study. Anesth Analg. 2022;134(1):149-158. doi:10.1213/ANE.0000000000005660 |

# Table S4. General characteristics and description of the 19 trials included in the systematic review.

| **№** | **Study** | **Journal** | **Sample size total** | **Sample size POD+** | **Sample size POD-** | **Design** | **Setting** | **POD assessment method** |
| --- | --- | --- | --- | --- | --- | --- | --- | --- |
| 1 | Abhijit V. Lele et al (2022) | J Neurosurg Anesthesiol | 112 | 10 | 102 | ROS | non-cardiac | CAM |
| 2 | Bradley A Fritz et al (2016) | Anesth Analg | 619 | 162 | 457 | POS | ND | CAM-ICU |
| 3 | Carolin Jung et al (2021) | Medicine (Baltimore) | 80 | 13 | 67 | POS | non-cardiac | 3D-CAM |
| 4 | Céline Khalifa et al (2023) | Eur J Anaesthesiol | 220 | 65 | 155 | POS | cardiac | CAM-ICU |
| 5 | Dana Baron Shahaf et al (2023) | J Cardiothorac Vasc Anesth | 772 | 48 | 724 | ROS | cardiac | CAM-ICU |
| 6 | Hirotaka Kinoshita et al (2023) | Anesth Analg | 80 | 25 | 55 | POS | non-cardiac | ICDSC |
| 7 | Juan C. Pedemonte et al (2020) | Anesthesiology | 159 | 23 | 136 | ROS | cardiac | CAM |
| 8 | Julian Ostertag et al (2023) | Anesthesiology | 169 | 32 | 137 | POS p-h | non-cardiac | CAM-ICU |
| 9 | Martin Soehle et al (2015) | BMC Anesthesiol | 81 | 26 | 55 | POS | cardiac | CAM-ICU |
| 10 | Melody Reese et al (2023) | Front Aging Neurosci | 83 | 12 | 71 | POS p-h | non-cardiac | CAM, 3D-CAM |
| 11 | Mona Momeni et al (2019) | J Clin Monit Comput | 1504 | 303 | 1201 | POS | cardiac | CAM |
| 12 | Na Li et al (2022) | Front Surg | 62 | 19 | 43 | POS | cardiac | CAM, CAM-ICU |
| 13 | Rieke Lutz et al (2022) | J Clin Anesth | 116 | 25 | 91 | POS | non-cardiac | CAM-ICU |
| 14 | S Hesse et al (2019) | Br J Anaesth | 626 | 125 | 501 | POS | non-cardiac | CAM-ICU |
| 15 | Srdjan Dragovic et al (2023) | Anesthesiology | 169 | 32 | 137 | ROS | non-cardiac | CAM-ICU |
| 16 | Susanne Koch et al (2023) | Front Aging Neurosci | 1058 | 198 | 860 | RCT p-h | non-cardiac | DSM IV |
| 17 | Susanne Koch et al (2021) | Anesth Analg | 237 | 41 | 196 | POS | non-cardiac | DSM V |
| 18 | Vera Röhr et al (2022) | Front Aging Neurosci | 1067 | 203 | 864 | RCT p-h | non-cardiac | DSM IV |
| 19 | Victoria Windmann et al (2022) | Front Aging Neurosci | 15 | 8 | 7 | POS | non-cardiac | DSM V |

**Abbreviations:** ROS, retrospective observational study; POS, prospective observational study; POS p-h, post hoc prospective observational study; RCT p-h, randomized controlled trial post hoc; POD, postoperative delirium; CAM, Confusion Assessment Method; DSM, diagnostic and statistical manual of mental disorders; ASA, American Society of Anesthesiologists; CAM-ICU, Confusion Assessment Method for the ICU; ICU, intensive care unit; 3D-CAM, 3-Minute Diagnostic Confusion Assessment Method; ICDSC, Intensive Care Delirium Screening Checklist; DSM, Diagnostic and Statistical Manual of Mental Disorders; ND, no data.

# Table S5. Patient and surgical characteristics of the 19 trials included in the review.

| **№** | **Study** | **Age** | **Age POD+** | **Age POD-** | **Sex, male (%)** | **Sex, % POD+** | **Sex, % POD-** | **ASA III-V, %** | **ASA III-V, % POD+** | **ASA III-V, % POD-** | **Anesthetic type** | **Surgery time, min POD+** | **Surgery time, min POD-** | **Anesthesia time, min POD+** | **Anesthesia time, min POD-** | **ICU stay, days POD+** | **ICU stay, days POD-** | **Hospital stay, days POD+** | **Hospital stay, days POD-** |
| --- | --- | --- | --- | --- | --- | --- | --- | --- | --- | --- | --- | --- | --- | --- | --- | --- | --- | --- | --- |
| 1 | Abhijit V. Lele et al (2022) | 59.8  (IQR:18.8) | 68  (IQR:19) | 58.5  (IQR:18) | 53.0 | 50 | 54.9 | ND | ND | ND | Propofol | 322 ± 248 | 341 ± 153 | ND | ND | 3.2 ± 6.4 | 2 ± 1.8 | 11.3 ± 16.8 | 5.2 ± 5.2 |
| 2 | Bradley A Fritz et al (2016) | 62 ± 14 | ND | ND | 64.0 | ND | ND | 61.9 | ND | ND | Sevoflurane  Desflurane  Isoflurane | ND | ND | ND | ND | ND | ND | ND | ND |
| 3 | Carolin Jung et al (2021) | ND | 70.68 ± 9.41 | 65.46 ± 10.61 | 58.8 | 69.2 | 56.7 | 45.0 | 46.2 | 44.8 | Sevoflurane | ND | ND | 234.59 ±  59.38 | 212.05 ±  79.21 | ND | ND | ND | ND |
| 4 | Céline Khalifa et al (2023) | ND | 74(64;79) | 67(59;74) | 81.8 | 80 | 82.6 | ND | ND | ND | Sevoflurane | 238 ± 60 | 226 ± 57 | ND | ND | 4 ± 3 | 2 ± 1 | 8(7;11) | 8(7;9) |
| 5 | Dana Baron Shahaf et al (2023) | ND | 71(66;75) | 66(56;72) | ND | ND | ND | ND | ND | ND | Isoflurane | 250  (207.5;292.5) | 240  (200;290) | ND | ND | ND | ND | ND | ND |
| 6 | Hirotaka Kinoshita et al (2023) | ND | 71.60 ± 7.77 | 64.98 ± 8.12 | 73.75 | 72 | 74.5 | 11.25 | 16 | 9.1 | Propofol | 546.6 ±  102.6 | 611.4 ±  133.2 | 616.2 ±  106.2 | 673.2 ± 145.8 | 4(4;6) | 4(4;4) | ND | ND |
| 7 | Juan C. Pedemonte et al (2020) | ND | 74(70;80) | 69(64;75) | 69 | ND | ND | 95.6 | 95.7 | 95.6 | Isoflurane | ND | ND | ND | ND | ND | ND | ND | ND |
| 8 | Julian Ostertag et al (2023) | ND | 69(61;77) | 59(48;73) | 75.1 | 81.3 | 73.7 | 23.7 | 43.7 | 19 | Sevoflurane Desflurane Propofol | ND | ND | 146.5  (99.50;184.50) | 81  (56.75;117.50) | ND | ND | ND | ND |
| 9 | Martin Soehle et al (2015) | 72.9 ± 6.2 | 74.5 ± 6.5 | 72.1 ± 5.9 | 70.4 | 61.5 | 74.5 | ND | ND | ND | Isoflurane | 321 ± 69 | 326 ± 74 | ND | ND | 3.375  (0.958;5.875) | 1.75  (0.83;3.75) | ND | ND |
| 10 | Melody Reese et al (2023) | 68(64;72) | 69.5  (64;77.5) | 67  (64;72) | 51.81 | 75 | 47.89 | 74.69 | 83.33 | 73.24 | Volatile  Propofol | ND | ND | ND | ND | ND | ND | ND | ND |
| 11 | Mona Momeni et al (2019) | 68(58;77) | 75  (64;80) | 67  (57;75) | 71 | ND | ND | ND | ND | ND | Sevoflurane/  Propofol | ND | ND | ND | ND | 3(2;5) | 2(2;3) | 10(7;14) | 7(7;10) |
| 12 | Na Li et al (2022) | ND | 64  (60;66) | 56  (51;62) | 64.5 | 57 | 67.0 | 100.0 | 100.0 | 100.0 | Propofol | 320  (229;441) | 260  (212;291) | 375(295;491) | 313(272;352) | ND | ND | 21(17;27) | 17(13;22) |
| 13 | Rieke Lutz et al (2022) | ND | 70  (65;77) | 59  (49;73) | 75.9 | 73.6 | 84 | 26.7 | 52 | 19.8 | Sevoflurane Desflurane  Propofol | ND | ND | 149(115;189) | 83(56;117) | ND | ND | ND | ND |
| 14 | S Hesse et al (2019) | ND | 63(50;71) | 56(44;67) | 61.0 | 59 | 61.0 | 48.2 | 45.3 | 60.0 | Sevoflurane Desflurane  Isoflurane  Propofol | ND | ND | 149(93;209) | 104(60;159) | ND | ND | 2(1;7) | 1(0;2) |
| 15 | Srdjan Dragovic et al (2023) | ND | 69(61;77) | 59(48;73) | 75.33 | 81 | 74 | 23.7 | 44 | 19 | Sevoflurane Desflurane  Propofol | ND | ND | 147(100;185) | 81(57;118) | ND | ND | ND | ND |
| 16 | Susanne Koch et al (2023) | 69.7 ± 6.3 | 72.1 ± 6.5 | 69.2 ± 6.1 | 54 | 53.5 | 55 | 47.7 | 60.6 | 44.8 | Sevoflurane Desflurane  Propofol | 235 ± 117 | 157 ± 92 | ND | ND | ND | ND | ND | ND |
| 17 | Susanne Koch et al (2021) | 72.8 ± 5.4 | 74.8 ± 5.4 | 72.3 ± 5.3 | 53 | 46 | 56 | 37 | 63.4 | 32 | Sevoflurane Desflurane Isoflurane  Propofol | ND | ND | 330 ± 180 | 192 ± 139 | ND | ND | ND | ND |
| 18 | Vera Röhr et al (2022) | ND | 71.9 ± 6.61 | 69.2 ± 6.06 | ND | ND | ND | ND | ND | ND | Sevoflurane Desflurane  Propofol | 232.8 ± 118.8 | 156.6 ± 91.8 | ND | ND | ND | ND | ND | ND |
| 19 | Victoria Windmann et al (2022) | 72(70;76) | 73(71;76) | 70(65;76) | 53.3 | 50 | 57.1 | 73.3 | 87.5 | 57.1 | Sevoflurane Desflurane  Propofol | 323(187;413) | 133(118;303) | ND | ND | 1.0(1.0;15.0) | 1.0(0.8;28) | 12.0(9.0;22.0) | 8(5.5;10.5) |

**Abbreviations:** POD, postoperative delirium; ICU, intensive care unit; CAM, Confusion Assessment Method; ASA, American Society of Anesthesiologists; ND, no data; IQR, interquartile range; Quantitative data are presented in median (Q1;Q3) or mean ± SD format.

# Table S6. EEG characteristics and outcome data of the 19 trials included in the review.

| **№** | **Study** | **Intraoperative EEG time point** | **EEG pattern** | **EEG pattern group** | **Value EEG pattern in POD+** | **Value EEG pattern in POD-** | **AUC (95% CI)** | **p value** |
| --- | --- | --- | --- | --- | --- | --- | --- | --- |
| 1 | Abhijit V. Lele et al (2022) | Intraoperatively | Burst suppression, n | Burst suppression | 100% | 67% | ND | ND |
|  |  |  | Length burst suppression, min | Burst suppression | 57.5 (IQR:87) | 45 (IQR:73) | ND | ND |
| 2 | Bradley A Fritz et al (2016) | Intraoperatively | Burst suppression, % of time | Burst suppression | Odds Ratio 1.22 (1.06 - 1.40) | | 0.62 (0.57; 0.67) | ND |
| 3 | Carolin Jung et al (2021) | Intraoperatively | Length burst suppression, min | Burst suppression | 27.09 ± 45.32 | 5.23 ± 10.80 | NA | 0.030 |
| 4 | Céline Khalifa et al (2023) | Intraoperatively | a centre of gravity (Hz) | Alpha | 9.85 ± 0.40 | 9.92 ± 0.38 | ND | 0.243 |
|  |  |  | b centre of gravity (Hz) | Beta | 16.84 ± 0.81 | 16.55 ± 0.76 | ND | 0.016 |
|  |  |  | d centre of gravity (Hz) | Delta | 1.89 ± 0.16 | 1.92 ± 0.13 | ND | 0.119 |
|  |  |  | Max a power (dB) | Alpha | -11.36 ± 5.28 | -8.85 ± 3.90 | ND | <0.001 |
|  |  |  | Max b power (dB) | Beta | -18.57 ± 3.83 | -16.18 ± 3.51 | ND | <0.001 |
|  |  |  | Max d power (dB) | Delta | -4.56 ± 3.05 | -3.41 ± 3.04 | ND | 0.010 |
|  |  |  | Max theta power (dB) | Theta | -13.37 ± 4.02 | -11.55 ± 3.67 | ND | 0.002 |
|  |  |  | Mean a power (dB) | Alpha | -14.03 ± 4.61 | -11.59 ± 3.37 | ND | <0.001 |
|  |  |  | Mean b power (dB) | Beta | -23.34 ± 3.27 | -21.16 ± 3.07 | ND | <0.001 |
|  |  |  | Mean d power (dB) | Delta | -9.65 ± 3.02 | -8.45 ± 3.01 | ND | 0.010 |
|  |  |  | Mean theta power (dB) | Theta | -14.94 ± 3.91 | -13.12 ± 3.48 | ND | 0.002 |
|  |  |  | Mean total power (dB) | Unclassified | -12.77 ± 2.88 | -11.68 ± 2.82 | ND | 0.004 |
|  |  |  | theta centre of gravity (Hz) | Theta | 5.93 ± 0.19 | 5.91 ± 0.20 | ND | 0.420 |
| 5 | Dana Baron Shahaf et al (2023) | Intraoperatively | Lateral interconnection ratio | Unclassified | 0.15 (0.02; 0.30) | -0.02 (-0.12; 0.08) | 0.779 (0.663; 0.895) | ≈0.001 |
| 6 | Hirotaka Kinoshita et al (2023) | Intraoperatively | Relative ratio of the α-power 3 h | Alpha | 0.15 ± 0.07 | 0.23 ± 0.10 | 0.749 (0.638; 0.860) | <0.001 |
|  |  |  | Relative ratio of the α-power 6 h | Alpha | 0.14 ± 0.06 | 0.22 ± 0.10 | 0.759 (0.653; 0.864) | <0.001 |
|  |  |  | Relative ratio of the α-power 9 h (or end) | Alpha | 0.13 ± 0.07 | 0.19 ± 0.08 | 0.728 (0.607; 0.849) | 0.003 |
|  |  |  | Relative ratio of the α-power baseline | Alpha | 0.18 ± 0.08 | 0.28 ± 0.11 | 0.776 (0.669; 0.883) | <0.001 |
| 7 | Juan C. Pedemonte et al (2020) | Intraoperatively | Alpha power, Hz | Alpha | 0.86 (0.46; 2.01) | 2.61 (1.26; 4.60) | ND | 0.001 |
|  |  |  | Burst suppression, n | Burst suppression | 65% | 33% | ND | ND |
| 8 | Julian Ostertag et al (2023) | Emergence | Alpha Power Absolute [dB] Aperiodic End | Alpha | -6.70 (-10.32; -4.29) | -6.04 (-8.06; -3.20) | 0.58 (0.48; 0.69) | 0.145 |
|  |  |  | Alpha Power Absolute [dB] Aperiodic Mid | Alpha | -7.27 (-9.83; -5.12) | -4.71 (-8.10; -1.82) | 0.67 (0.58; 0.77) | 0.002 |
|  |  |  | Alpha Power Absolute [dB] Aperiodic Start | Alpha | -7.02 (-10.71; -5.86) | -4.32 (-7.24; -0.14) | 0.70 (0.61; 0.79) | 0.001 |
|  |  |  | Alpha Power Absolute [dB] Conventional End | Alpha | 1.44 (-1.39; 5.37) | 5.11 (0.80; 9.15) | 0.65 (0.55; 0.74 | 0.011 |
|  |  |  | Alpha Power Absolute [dB] Conventional Mid | Alpha | 2.29 (-2.20; 6.80) | 8.68 (4.69; 11.93) | 0.720 (0.63; 0.81) | <0.001 |
|  |  |  | Alpha Power Absolute [dB] Conventional Start | Alpha | 0.31 (-3.74; 7.63) | 8.84 (4.04; 12.81) | 0.74 (0.65; 0.82) | <0.001 |
|  |  |  | Alpha Power Absolute [dB] Periodic End | Alpha | 7.99 (5.97; 9.54) | 9.63 (7.10; 12.13) | 0.65 (0.55; 0.75 | 0.007 |
|  |  |  | Alpha Power Absolute [dB] Periodic Mid | Alpha | 8.37 (5.13; 11.15) | 11.92 (9.54; 14.15) | 0.74 (0.65; 0.82) | <0.001 |
|  |  |  | Alpha Power Absolute [dB] Periodic Start | Alpha | 8.30 (5.10; 11.05) | 11.99 (9.18; 13.58) | 0.72 (0.63; 0.81) | <0.001 |
|  |  |  | Alpha Power Relative [%] Aperiodic End | Alpha | 11.72 (10.21; 13.22) | 11.78 (9.70; 13.58) | 0.50 (0.39; 0.61) | 0.957 |
|  |  |  | Alpha Power Relative [%] Aperiodic Mid | Alpha | 10.11 (8.54; 11.57) | 9.63 (7.85; 11.35) | 0.45 (0.34; 0.56) | 0.383 |
|  |  |  | Alpha Power Relative [%] Aperiodic Start | Alpha | 9.70 (7.59; 11.56) | 8.79 (6.85; 10.64) | 0.41 (0.30; 0.53) | 0.122 |
|  |  |  | Alpha Power Relative [%] Conventional End | Alpha | 22.88 (17.98; 34.10) | 31.46 (22.38; 40.15) | 0.65 (0.55; 0.75) | 0.010 |
|  |  |  | Alpha Power Relative [%] Conventional Mid | Alpha | 26.53 (16.99; 34.46) | 34.24 (28.04; 43.00) | 0.70 (0.61; 0.79) | 0.001 |
|  |  |  | Alpha Power Relative [%] Conventional Start | Alpha | 22.74 (17.32; 35.21) | 31.66 (22.95; 40.49) | 0.67 (0.58; 0.77) | 0.003 |
|  |  |  | Alpha Power Relative [%] Periodic End | Alpha | 21.65 (16.31; 28.52) | 26.28 (19.22; 34.55) | 0.62 (0.52; 0.72) | 0.039 |
|  |  |  | Alpha Power Relative [%] Periodic Mid | Alpha | 21.24 (16.48; 31.01) | 31.34 (23.86; 38.65) | 0.71 (0.62; 0.80) | <0.001 |
|  |  |  | Alpha Power Relative [%] Periodic Start | Alpha | 21.98 (17.43; 28.83) | 31.38 (25.29; 37.60) | 0.72 (0.63; 0.81) | <0.001 |
|  |  |  | Burst suppression, n | Burst suppression | 25% | 9% | ND | ND |
| 9 | Martin Soehle et al (2015) | Intraoperatively | Burst suppression ratio, % | Burst suppression | 1.24 (0.30; 3.34) | 0.44 (0.06; 2.00) | 0.68 (ND) | ND |
|  |  |  | EEG asymmetry | Unclassified | ND | ND | 0.63 (ND) | ND |
|  |  |  | Length burst suppression, min | Burst suppression | 107 (47; 170) | 44 (11; 120) | 0.73 (ND) | ND |
| 10 | Melody Reese et al (2023) | Intraoperatively | Burst suppression, n | Burst suppression | 33% | ND | ND | ND |
|  |  |  | preBurst suppression | Burst suppression | 100% | 100% | ND | ND |
|  |  |  | preBurst suppression, % min | Burst suppression | 100% | 100% | ND | ND |
| 11 | Mona Momeni et al (2019) | Intraoperatively | AUCEEGSR>0s (AUC of EEG suppression ratio), min% | Burst suppression | ND | ND | NA | <0.001 |
|  |  |  | Burst suppression ratio, % | Burst suppression | ND | ND | NA | <0.001 |
| 12 | Na Li et al (2022) | Intraoperatively | epileptiform discharges | Unclassified | 53% | 14% | NA | ND |
| 13 | Rieke Lutz et al (2022) | Intraoperatively | Burst suppression, n | Burst suppression | 76% | 71% | ND | ND |
|  |  |  | absolute alpha power, dB | Alpha | ND | ND | 0.77 (0.660.87) | ND |
|  |  |  | Burst suppression maintenance | Burst suppression | 52% | 21% | ND | ND |
|  |  |  | Burst suppression peri-induction | Burst suppression | 48% | 62% | ND | ND |
|  |  |  | fronto-parietal coherence | Unclassified | ND | ND | 0.71 (0.590.82) | ND |
|  |  |  | relative alpha power, dB | Alpha | ND | ND | 0.71 (0.570.83) | ND |
| 14 | S Hesse et al (2019) | Intraoperatively | Burst suppression, n | Burst suppression | 44% | 37% | ND | ND |
| 15 | Srdjan Dragovic et al (2023) | Intraoperatively | Alpha Power or EEG Falling, n | Alpha | 47% | 74% | ND | ND |
|  |  |  | Alpha Power or EEG Not changing, n | Alpha | 6% | 7% | ND | ND |
|  |  |  | Alpha Power or EEG Rising, n | Alpha | 47% | 19% | ND | ND |
|  |  |  | Alpha band power, µV2/Hz | Alpha | ND | ND | 0.67 (0.56; 0.77) | ND |
|  |  |  | Starting Alpha power, µV2/Hz | Alpha | ND | ND | 0.26 (0.16; 0.37) | ND |
|  |  |  | Beta Power or EEG Falling, n | Beta | 28% | 56% | ND | ND |
|  |  |  | Beta Power or EEG Not changing, n | Beta | 6% | 9% | ND | ND |
|  |  |  | Beta Power or EEG Rising, n | Beta | 66% | 34% | ND | ND |
|  |  |  | Beta band power, µV2/Hz | Beta | ND | ND | 0.67 (0.58; 0.76) | ND |
|  |  |  | Delta Power or EEG Falling, n | Delta | 41% | 63% | ND | ND |
|  |  |  | Delta Power or EEG Not changing, n | Delta | 6% | 7% | ND | ND |
|  |  |  | Delta Power or EEG Rising, n | Delta | 53% | 30% | ND | ND |
|  |  |  | Starting Beta power, µV2/Hz | Beta | ND | ND | 0.33 (0.21; 0.45) | ND |
|  |  |  | Delta band power, µV2/Hz | Delta | ND | ND | 0.64 (0.52; 0.74) | ND |
|  |  |  | Starting Delta power, µV2/Hz | Delta | ND | ND | 0.32 (0.22; 0.43) | ND |
|  |  |  | Starting Theta power, µV2/Hz | Theta | ND | ND | 0.31 (0.21; 0.41) | ND |
|  |  |  | Starting total power, µV2/Hz | Unclassified | ND | ND | 0.29 (0.20; 0.40) | ND |
|  |  |  | Theta band power, µV2/Hz | Theta | ND | ND | 0.68 (0.58; 0.77) | ND |
|  |  |  | Theta Power or EEG Falling, n | Theta | 63% | 77% | ND | ND |
|  |  |  | Theta Power or EEG Not changing, n | Theta | 3% | 6% | ND | ND |
|  |  |  | Theta Power or EEG Rising, n | Theta | 34% | 17% | ND | ND |
|  |  |  | Total band power, µV2/Hz | Unclassified | ND | ND | 0.67 (0.56; 0.77) | ND |
|  |  |  | Total Power or EEG Falling, n | Unclassified | 38% | 66% | ND | ND |
|  |  |  | Total Power or EEG Not changing, n | Unclassified | 13% | 11% | ND | ND |
|  |  |  | Total Power or EEG Rising, n | Unclassified | 50% | 23% | ND | ND |
| 16 | Susanne Koch et al (2023) | Intraoperatively | Burst suppression ratio | Burst suppression | 0.2 ± 0.11 | 0.19 ±0.1 | NA | n.s. |
|  |  |  | Length burst suppression, min | Burst suppression | 27.5 ± 21.3 | 21.4 ± 16.2 | NA | < 0.001 |
| 17 | Susanne Koch et al (2021) | Intraoperatively | Spectral edge frequency intraoperative, Hz | Unclassified | 12.4 ± 5.2 | 12.8 ± 3.2 | NA | 0.564 |
|  |  |  | Spectral edge frequency postinduction, Hz | Unclassified | 12.4 ± 5.5 | 12.7 ± 3.9 | NA | 0.739 |
|  |  |  | Length burst suppression, min | Burst suppression | median: 32 (95%CI: 7; 200) | median: 17 (95%CI: 16; 39) | NA | 0.227 |
| 18 | Vera Röhr et al (2022) | Intraoperatively | Burst suppression ratio | Burst suppression | 0.197 ± 0.1 | 0.193 ± 0.1 | ND | ND |
| 19 | Victoria Windmann et al (2022) | Intraoperatively | DC-shifts (changes in the direct current (DC) EEG), μV/s | Unclassified | 31.6 (22.7; 38.9) | 4.7 (2.2; 12.5) | NA | 0.026 |

**Abbreviations:** POD, postoperative delirium; EEG, electroencephalogram; AUC, area under the ROC curve; ROC, receiver operating characteristic; ND, no data; CI, confidence interval.

# Table S7. Certainty of evidence for studied outcomes (Grade approach).

| Outcome | No. of participants and studies | Risk of  bias | Inconsistency | Indirectness | Imprecision | Publication bias | Upgrades | Overall  quality of evidence |
| --- | --- | --- | --- | --- | --- | --- | --- | --- |
| Presence of burst suppression  Statement: Presence of burst suppression is associated with increased risk of postoperative delirium in adults. OR = 1.68 (1.22; 2.32), RR = 1.41 (1.1; 1.8) | 1,182,  5 studies | Serious  (-1) | Not serious (0) | Not serious (0) | Serious  (-1) | Serious  (-1) | No | ⊕ΟΟΟ  Very low |
| Duration of burst suppression  Statement: Increased duration of burst suppression is associated with increased risk of postoperative delirium in adults. MD = 15.86 (3.02; 28.70) minutes; SMD = 0.36 (0.23; 0.49) | 1,568,  5 studies | Serious  (-1) | Not serious (0) | Not serious (0) | Not serious (0) | Not serious (0) | No | ⊕⊕⊕Ο  Moderate |
| Burst suppression ratio  Statement: Burst suppression ratio is not associated with risk of postoperative delirium in adults. MD = 0.007 (-0.004; 0.018); SMD = 0.07 (-0.04; 0.18) | 2,125,  2 studies | Serious  (-1) | Not serious (0) | Not serious (0) | Not serious (0) | Not serious (0) | No | ⊕⊕⊕Ο  Moderate |

**Abbreviations:** OR, odds ratio; RR, relative risk; CI, confidence interval; SMD, standardized mean difference.

Key: 0, no evidence downgrade; -1, serious limitation; -2, very serious limitation; +1, evidence upgrade.

The baseline evidence level for studies of prognostic factors was high.

# Figure S1. Forest plot for the presence of burst suppression (OR).


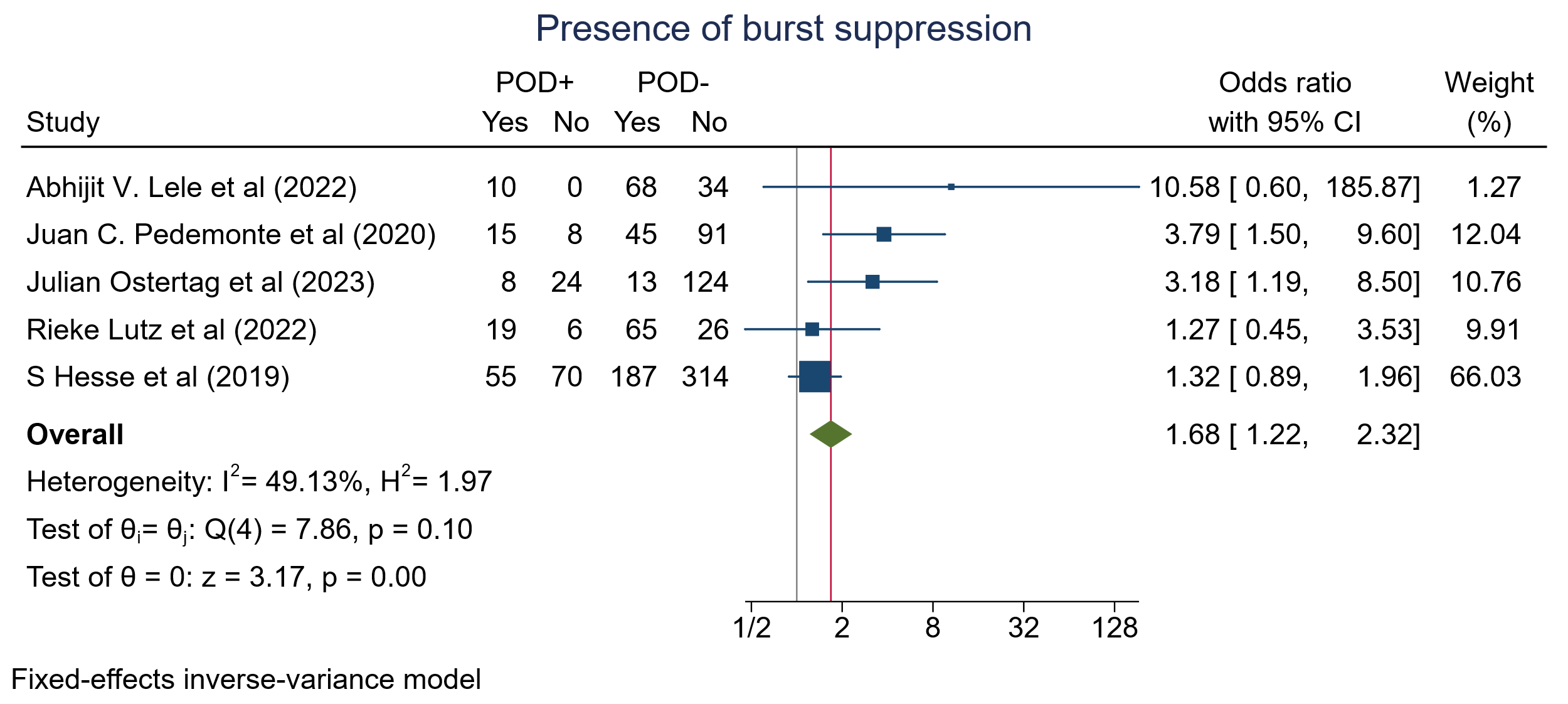


The plot displays the studies, sample size, odds ratio (OR), confidence interval (CI), and p value. The size of the squares indicates the weight of the studies (considering sample size and standard deviations); the diamond represents the pooled OR with CIs.

# Figure S2. Forest plot for the presence of burst suppression (log OR)


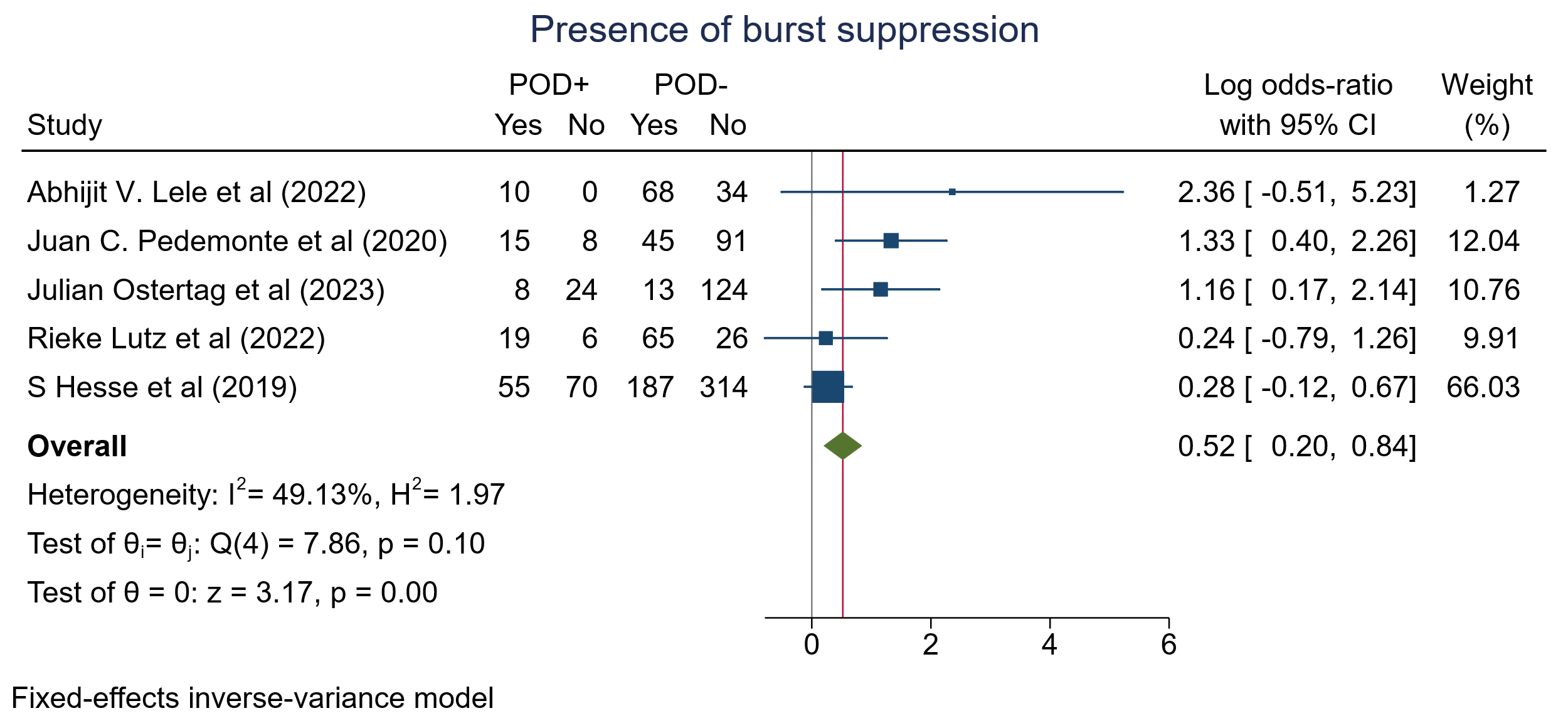


The plot displays the studies, sample size, log-transformed odds ratio (log OR), confidence interval (CI), and p value. The size of the squares indicates the weight of the studies (considering sample size and standard deviations); the diamond represents the pooled Log OR with CIs.

# Figure S3. Forest plot for the presence of burst suppression (RR)


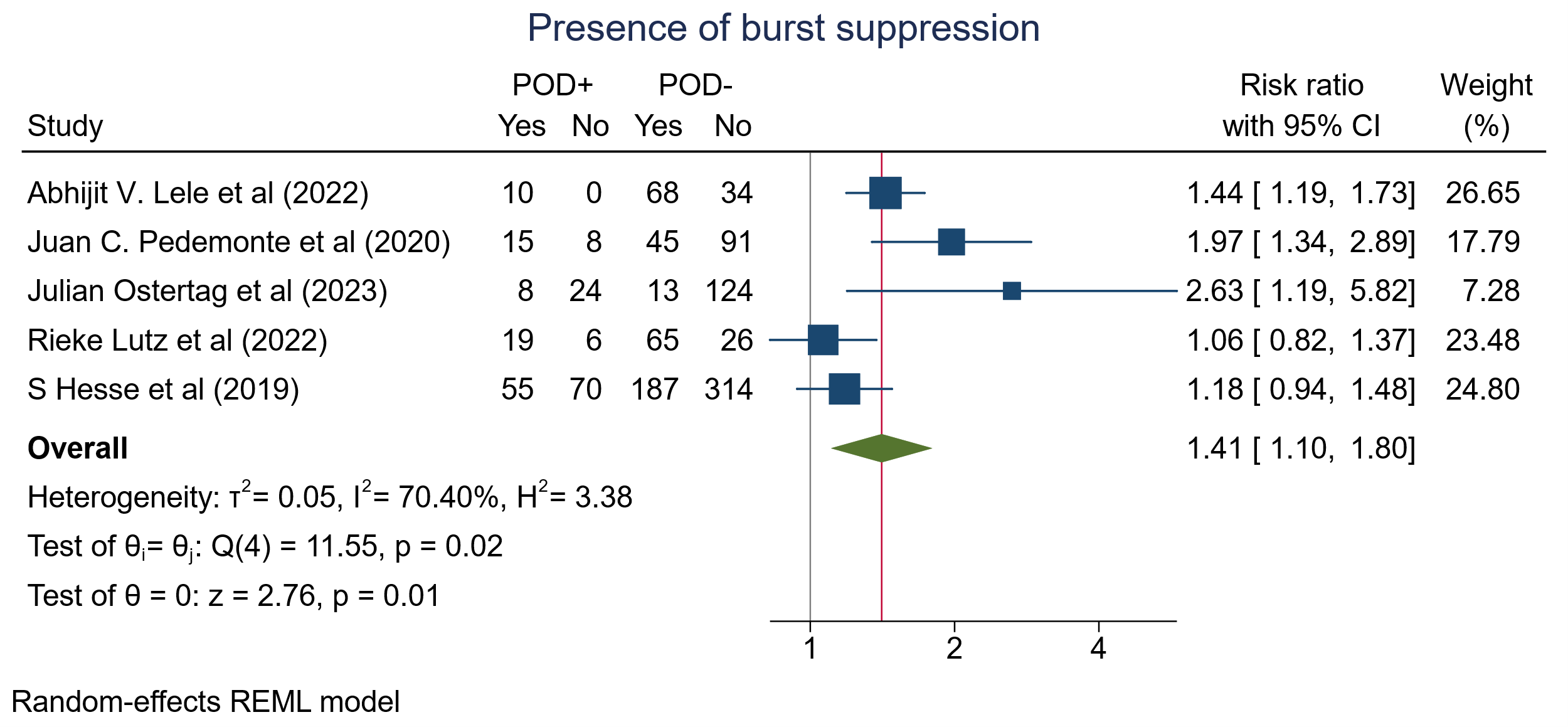


The plot displays the studies, sample size, risk ratio (RR), confidence interval (CI), and p value. The size of the squares indicates the weight of the studies (considering sample size and standard deviations); the diamond represents the pooled RR with CIs.

# Figure S4. Forest plot of sensitivity and specificity for the presence of burst suppression.


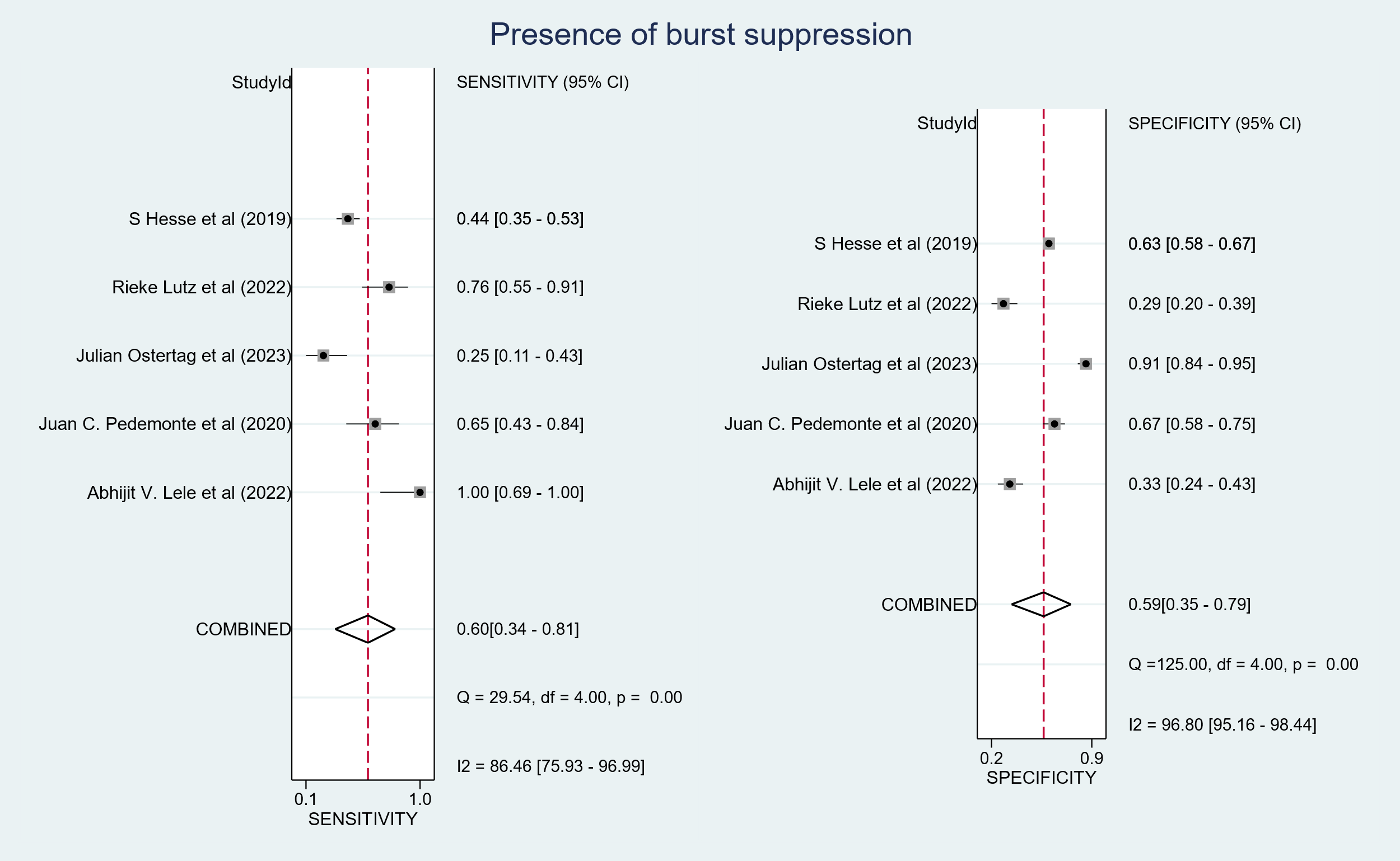


The plot displays the studies, sensitivity and specificity, 95% confidence intervals (CIs), and p values.

# Figure S5. Forest plot for duration of burst suppression (MD).


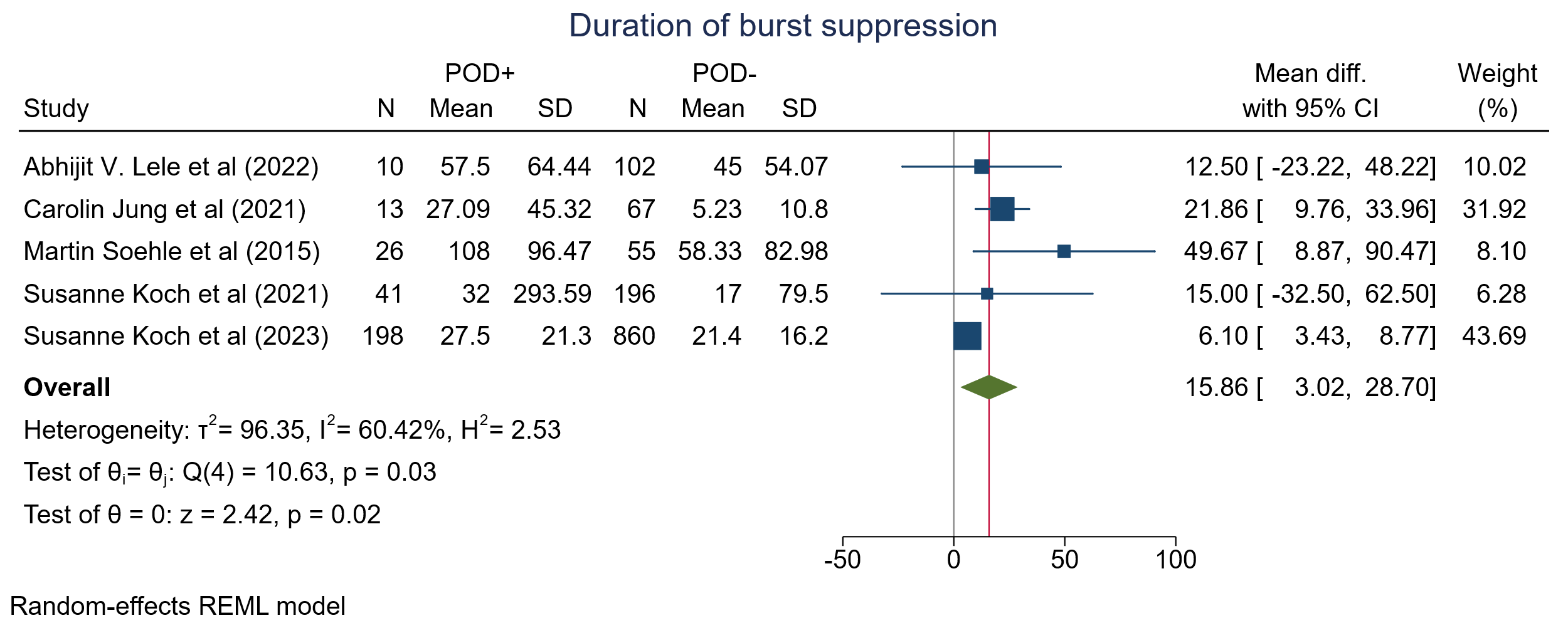


The plot displays the studies, sample size, mean difference (MD), confidence interval (CI), and p value.

The size of the squares indicates the weight of the studies (considering sample size and standard deviations); the diamond represents the pooled MD with CIs.

# Figure S6. Forest plot for duration of burst suppression (SMD).


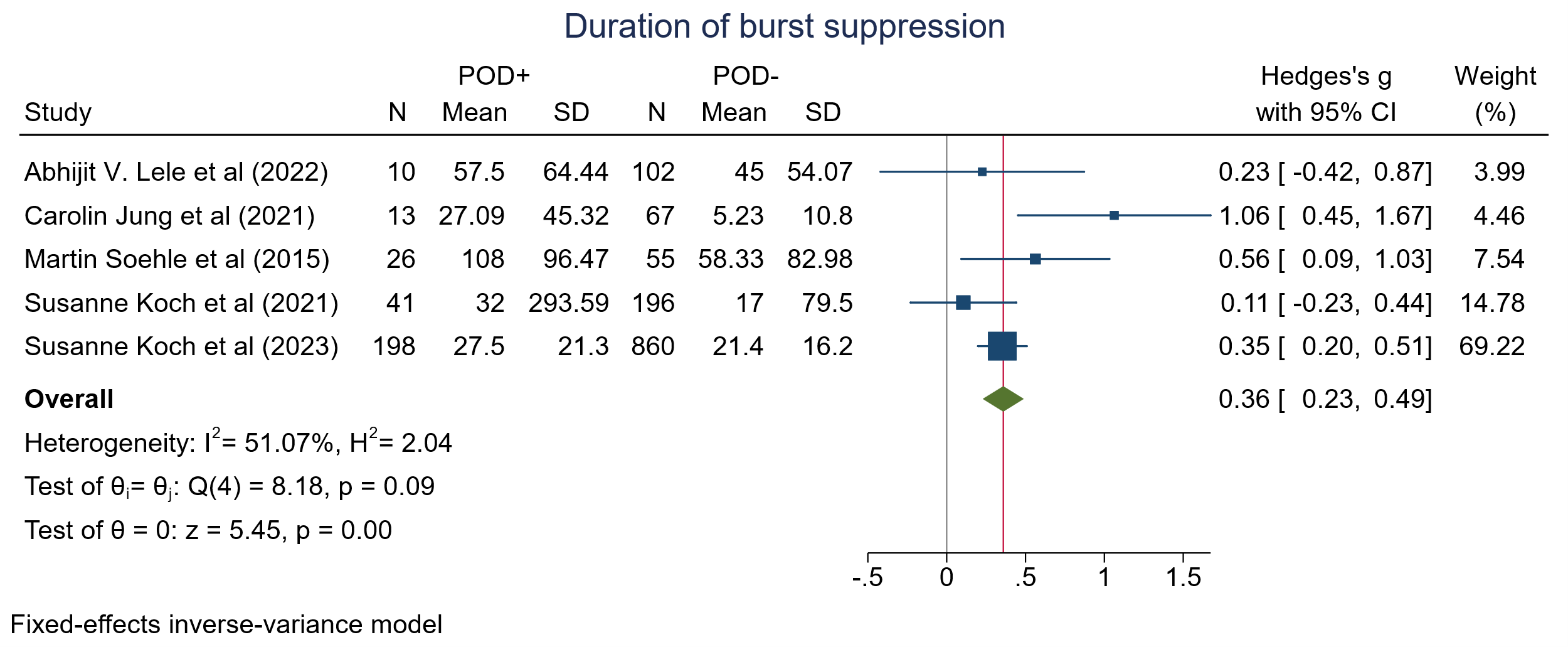


The plot displays the studies, sample size, standardized mean difference (SMD), confidence interval (CI), and p value.

The size of the squares indicates the weight of the studies (considering sample size and standard deviations); the diamond represents the pooled SMD with CIs.

# Figure S7. Forest plot for the burst suppression ratio (MD).


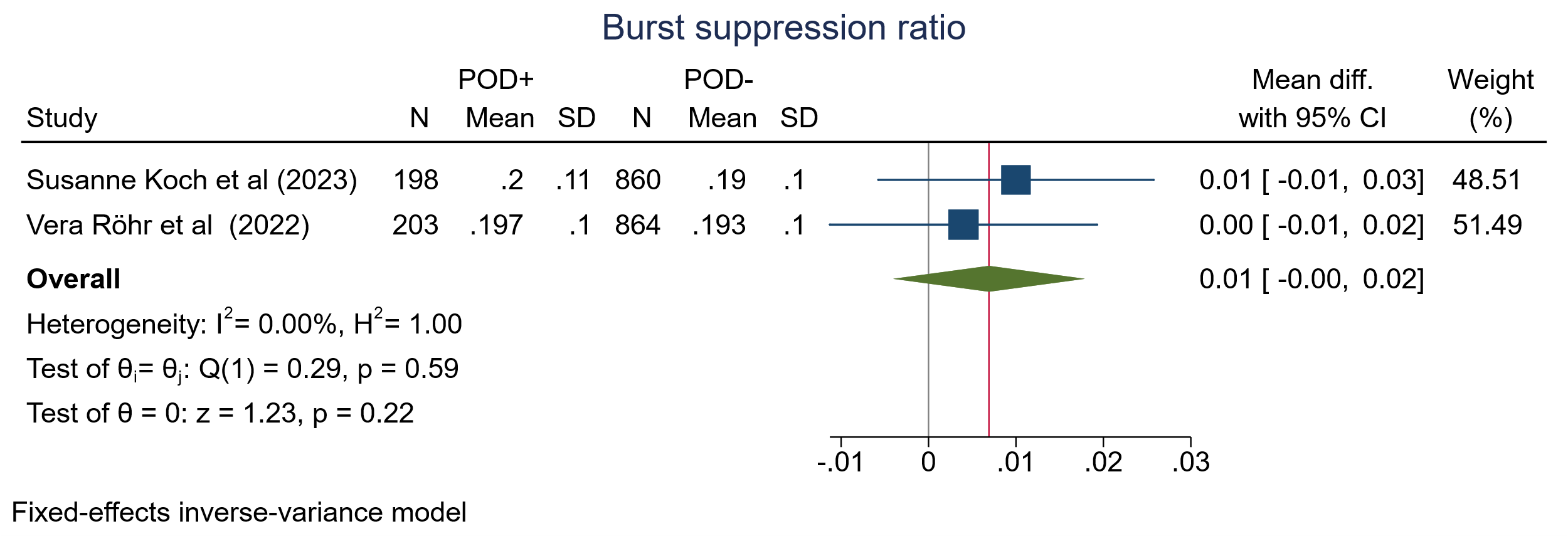


The plot displays the studies, sample size, mean difference (MD), confidence interval (CI), and p value. The size of the squares indicates the weight of the studies (considering sample size and standard deviations); the diamond represents the pooled MD with CIs.

# Figure S8. Forest plot for the burst suppression ratio (SMD).


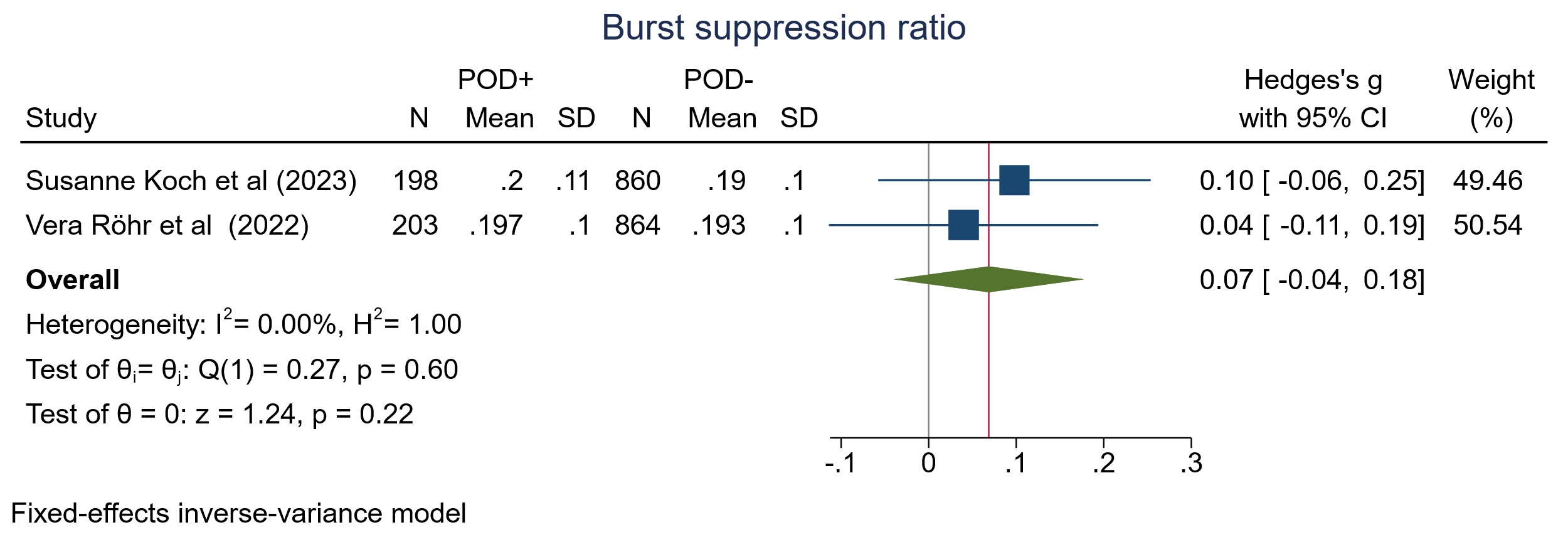


The plot displays the studies, sample size, standardized mean difference (SMD), confidence interval (CI), and p value. The size of the squares indicates the weight of the studies (considering sample size and standard deviations); the diamond represents the pooled SMD with CIs.

# Figure S9. Risk of bias evaluation of the included trials using the “Tool to assess risk of bias in cohort studies” by the CLARITY Group at McMaster University.

**Abbreviations:** DY, Definitely Yes; PY, Probably Yes; PN, Probably No; DN, Definitely No.

# Figure S10. Funnel plots.

Presence of burst suppression:


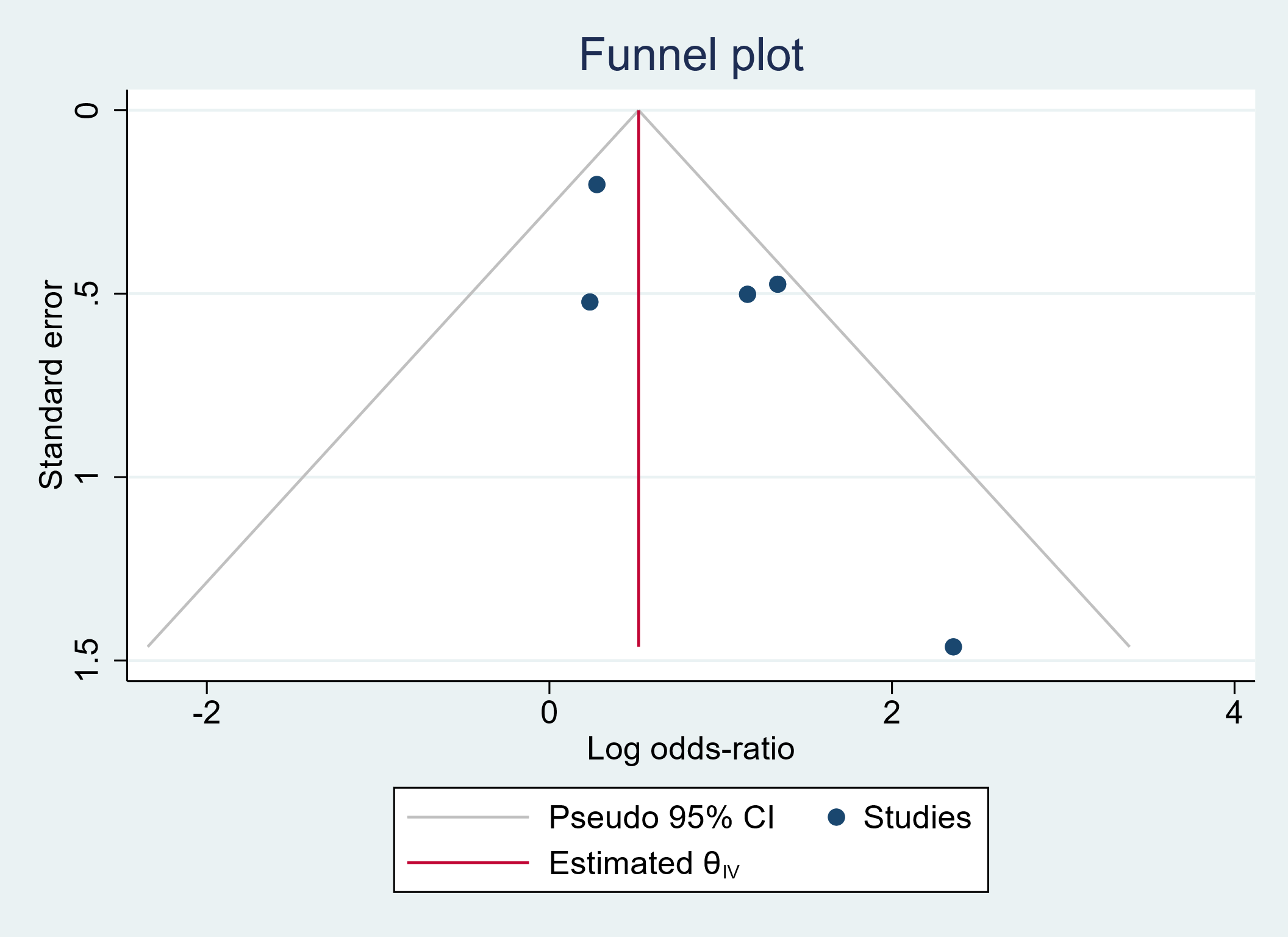


Duration of burst suppression:


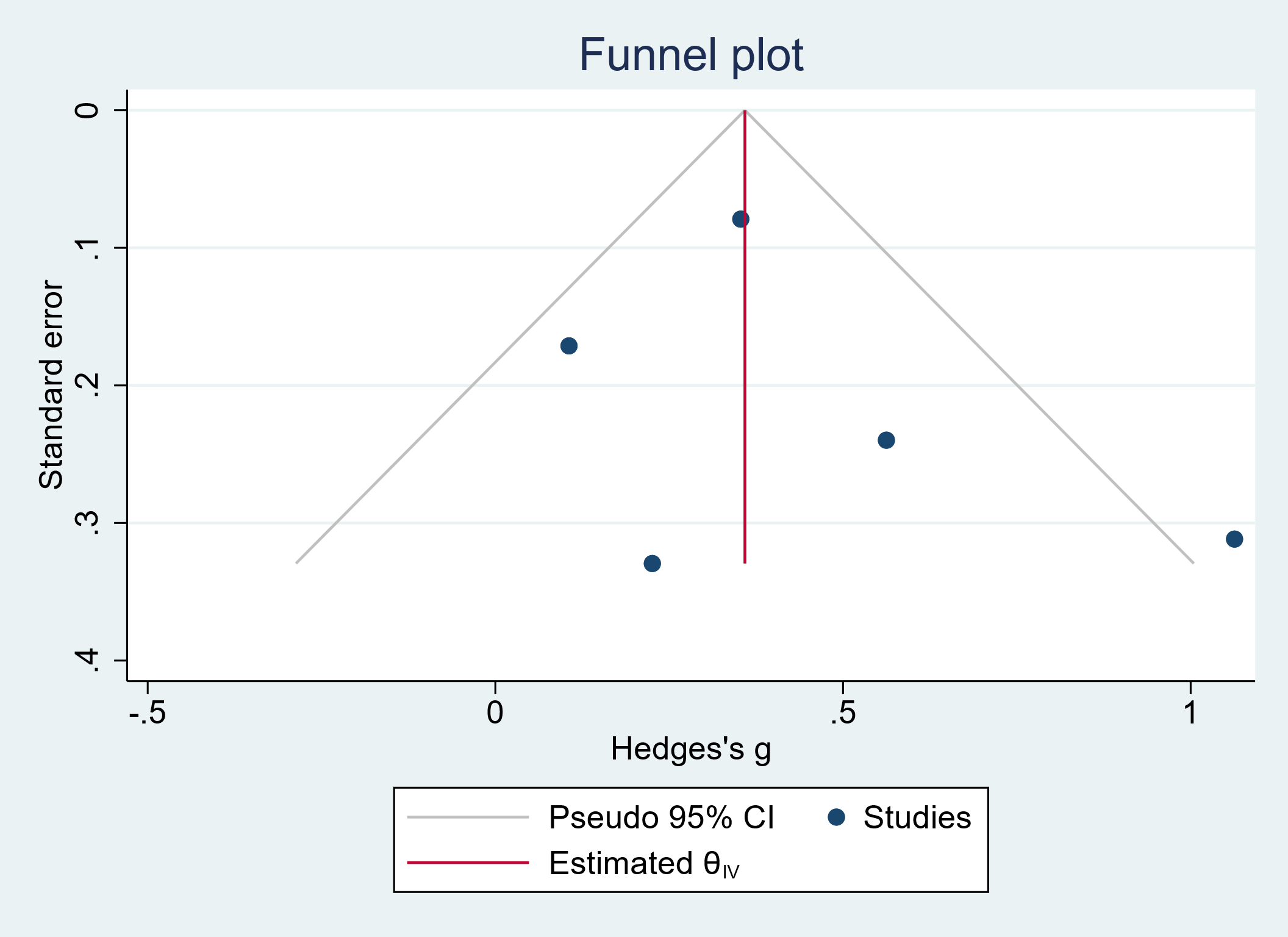


Burst suppression ratio:

**
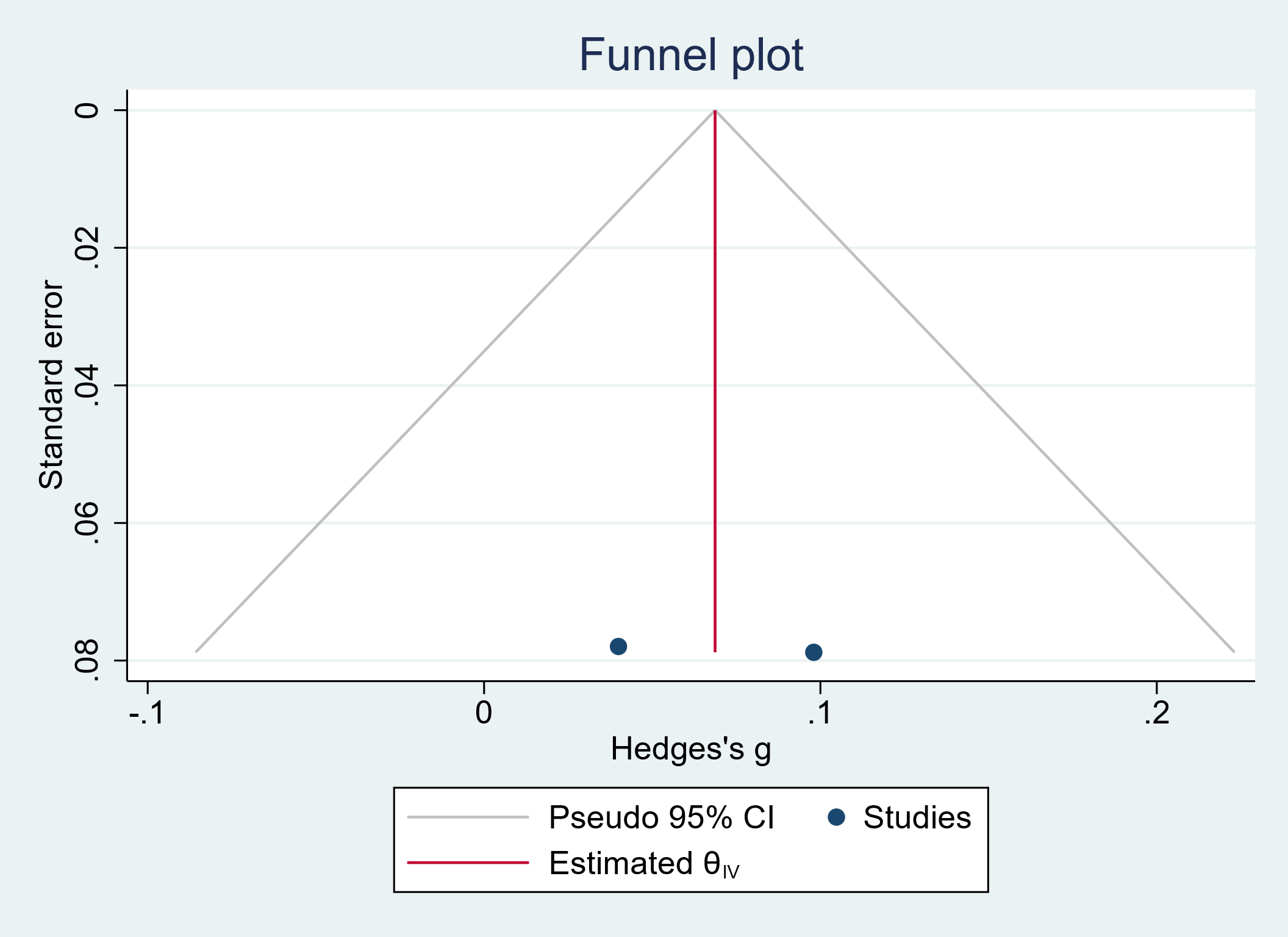
**
